# P140 Peptide Leads to Clearance of Autoreactive Lymphocytes and Normalizes Immune Response in Lupus-Prone Mice

**DOI:** 10.3389/fimmu.2022.904669

**Published:** 2022-06-01

**Authors:** Nicolas Schall, Laura Talamini, Maud Wilhelm, Evelyne Jouvin-Marche, Sylviane Muller

**Affiliations:** ^1^ CNRS and Strasbourg University, Unit Biotechnology and Cell signaling, UMR7242/Strasbourg Drug Discovery and Development Institute (IMS), Strasbourg, France; ^2^ Institute for Advanced Biosciences, Research Centre Université Grenoble Alpes (UGA)-Inserm U1209-CNRS UMR 5309, La Tronche, France; ^3^ Fédération Hospitalo-Universitaire (FHU) OMICARE, Fédération de Médecine Translationnelle de Strasbourg (FMTS), Strasbourg University, Strasbourg, France; ^4^ University of Strasbourg Institute for Advanced Study, Strasbourg, France

**Keywords:** lupus, antigen-presenting cells, class II MHC molecules, TCR/BCR, autophagy, P140 peptide-based treatment

## Abstract

In systemic lupus erythematosus, T cells display multiple abnormalities. They are abnormally activated, secrete pro-inflammatory cytokines, help B cells to generate pathogenic autoantibodies, and provoke the accumulation of autoreactive memory T cells. P140, a synthetic peptide evaluated in phase-III clinical trials for lupus, binds HSPA8/HSC70 chaperone protein. *In vitro* and *in vivo*, it interferes with hyperactivated chaperone-mediated autophagy, modifying overexpression of major histocompatibility complex class II molecules and antigen presentation to autoreactive T cells. Here, we show that in P140-treated lupus mice, abnormalities affecting T and B cells are no longer detectable in secondary lymphoid tissue and peripheral blood. Data indicate that P140 acts by depleting hyper-activated autoreactive T and B cells and restores normal immune homeostasis. Our findings suggest that P140 belongs to a new family of non-immunosuppressive immunoregulators that do not correct T and B cell abnormalities but rather contribute to the clearance of deleterious T and B cells.

## Introduction

Systemic lupus erythematosus (SLE) is a complex autoimmune disorder predominantly affecting young female population. It is characterized by unpredictable exacerbation and remission phases, hypocomplementemia and autoantibodies directed mostly against nuclear antigens (1, 2). Chronic inflammation occurring in SLE can virtually affect any organs making this syndrome highly polymorphic and sometimes difficult to diagnose (3–5). Successful genome wide association studies have shown that both common gene polymorphisms and rare genetic variants can both contribute to SLE susceptibility (6, 7). A polygenic predisposition, sex hormones, and environmental triggers are known drivers of the disordered immune response that typifies the disease. Both T and B cells are involved in the multiple, generally interconnected immunoregulatory abnormalities, which characterize lupus (1, 8–10). Solid evidence supports the notion that defects in their signaling and an abnormal skewing of the cytokine production contribute to the pathophysiology. The disturbed T cell biology in lupus is central (11). Functional abnormalities of T cells are notable in all T cell subtypes, including T helper (TH)1, TH2 and TH17 cells, T follicular helper CD4^+^ cells, γδ T cells [a subset of them expressing Vδ2 is reduced in peripheral blood but accumulates in kidneys from SLE patients (12)]. A population of mature double negative (DN) T cells likely arising from activated CD8^+^ T cells and defined as T cell receptor (TCR)αβ^+^CD3^+^CD4^−^CD8^−^B220/CD45R^+^ accumulate and produce pro-inflammatory cytokine IL-17 (13). This unique T cell compartment is particularly expanded in Fas (CD95/APO-1)-deficient Murphy Roths large (MRL)/lymphoproliferation (lpr) lupus-prone mice that develop massive lymphadenopathy associated with proliferation of aberrant T cells. Abnormalities also affect several subtypes of regulatory T (Treg) cells, including TCRαβ CD4^+^, TCRαβ CD8^+^ and γδ Treg cells. IL-17-producing T cells (TH17, γδ and DN T cells) amplify renal impairment by supporting self-reactive B-cell survival, differentiation, and subsequent antibody production, and by promoting inflammation in infiltrated tissues (14, 15).

Some years ago, we discovered a phosphopeptide, P140, which behaves as a potent non-immunosuppressive regulator of the autoimmune response. This synthetic 21-mer linear peptide initially identified from a cellular screen of overlapping peptides covering the small nuclear ribonucleoprotein U1-70K (16), exhibits protective activity in MRL/lpr mice and patients with SLE (17–20). In a multicenter, randomized, placebo-controlled phase-IIb clinical trial, P140/Lupuzor had no adverse safety signals in SLE patients and met its primary efficacy end points (19, 20). This peptide is stable and non-immunogenic in mice and patients (21). Our previous studies showed that P140 displays its protective effects *via* a mechanism that involves autophagy (22–26). Abnormalities in the autophagy pathway have been identified both in T and B cells from lupus patients (27–30). Naturally-occurring immunoglobulin (Ig)G antibodies reacting with lysosomal-associated membrane protein 2A (LAMP2A) have been characterized both in mice and patients with SLE (31). LAMP2A, in contrast to other spliced protein variants of the *Lamp2* gene, is considered as a rate-limiting factor in the lysosomal degradation stage of autophagy; it plays a pivotal trafficking role in chaperone-mediated autophagy (CMA) by allowing the translocation across the lysosomal membrane of cytosolic proteins targeted by heat shock protein 8 (HSPA8)/HSC70 (32). In lupus T cells, and in naïve CD4^+^ T cells in particular, autophagic vacuoles are more abundant and autophagosome-associated microtubule-associated protein light chain 3 (MAP1LC3-II) isoform is over-expressed, indicating that macroautophagy is hyperactivated (27, 29). Autophagy appears particularly activated in naïve B cell subsets, and when autophagy inhibitors were used, plasmablast (PB) differentiation and survival hardly occurred (29). Upon treatment of MRL/lpr mice with P140, the abnormal expression of several autophagy markers returns to baseline level, reflecting potent effect of P140 on this process. Especially, the expression of autophagy markers sequestosome 1 (SQSTM1)/p62 and MAP1LC3 was corrected in B cells, indicating that excessive autophagic flux level was restored (22). Overexpression in MRL/lpr B cells of HSPA8, to which P140 readily interacts (23), and LAMP2A was globally down-regulated (22, 24). A range of alterations affecting lysosomes were no longer detectable (26). We have discovered that in MRL/lpr mice, the effect of P140 occurs at the step of substrate lysosomal uptake (26).

P140 uses the clathrin-dependent endo-lysosomal pathway to enter into MRL/lpr B lymphocytes and accumulates in the lysosomal lumen (24). Consistent with our experimental data, we proposed that within lysosome, P140 encounters and inhibits lysosomal HSP90AA1 and HSPA8, which are responsible of the assembly of LAMP2A multiplex and translocation of CMA substrates, respectively (26). P140 alters the composition of HSPA8 heterocomplexes and directly hampers HSPA8 chaperoning properties (22, 24) that are known in the context of autophagy to be decisive in antigen processing for major histocompatibility complex class II (MHCII) presentation (33–37). Upon P140 treatment (*in vivo* in MRL/lpr mice and *ex vivo* in SLE patients), we effectively observed a lower expression of MHCII molecules in antigen-presenting cells (APCs) that are mostly B cells in lupus (38), a weaker activity of autoreactive CD4^+^ T cells, and a lower number of plasma cells (17, 22, 25, 39, 40). A drop of autoantibody reactivity to double-stranded deoxyribonucleic acid (dsDNA) was found in the peripheral blood collected from patients (19). In the MRL/lpr mouse model, lupus-like disease correlates with proteinuria, an indicator of renal failure, and high anti-dsDNA antibody serum levels. Both were attenuated upon treatment with P140, as well as IgG antibodies to Ro52/TRIM1, with a prolongation of survival of P140 treated MRL/lpr mice (17, 18, 25). P140 diminished the extent of dermatitis, and vasculitis with less perivascular inflammatory infiltrates (17, 22). No effect was measured using saline or the non-phosphorylated peptide 131–151 as control treatment.

Even though we know that P140/Lupuzor exerts efficient therapeutic effects in mice and patients with lupus, virtually nothing is known about its capacity to reconstitute immune tolerance in treated MRL/lpr mice and especially how it can recover abnormalities of T and B cells. To address these questions, we compared several key cellular and molecular elements of the MRL/lpr autoimmune response with healthy MHC-matched CBA/J mice (trafficking properties of T and B cells, TCR and BCR repertoires of peripheral blood mononuclear cells (PBMCs) and splenocytes, ability of immune cells to secrete soluble cytokines, capacity of the immune system to mount a response to an exogenous antigen, ability of plasma cells (PCs) to secrete Ig). To complete the picture, experiments were also performed with defective MRL/lpr mice that spontaneously exhibit T cell impairment. Collectively, an unexpected finding emerged from these studies: *via* its effect on CMA and antigen presentation by APCs, P140 contributes to the clearance - not to an immune diversion - of pathology-associated lymphocyte compartments, thereby limiting the functional activity of potentially self-reactive CD4^+^ T and B cells.

## Materials and Methods

### Mice, Treatments and Classical Disease Monitoring Tests

Female CBA/J (cross between a Bagg albino female and a DBA male), C57BL/6 (cross between a female N.57 and a male N.52 from the A. Lathrop’lab) and MRL/lpr mice (composite genome derived 75.0% from LG/J, 12.6% AKR/J, 12.1% C3H/HeDi and 0.3% C57BL/6J) were purchased from Harlan-France or Charles River-France/Jackson Laboratory. Genotyping of mice (*Fas* gene) was done using the primers and PCR protocols provided by The Jackson laboratories for MRL/lpr mice. In most of experiments including those conducted to explore TCR and BCR V-J rearrangements, 11-13 week-old female MRL/lpr mice received a single intravenous (i.v.) administration of P140 peptide in saline or saline alone as control. MHC-matched CBA/J mice were used as healthy control. For testing the effect of P140 on the ability of MRL/lpr mice to mount an immune response to a foreign immunogen, 14 week-old female CBA/J and MRL/lpr mice received P140 at weeks 5, 7, 9, and 13 (100µg in 100µL saline per mouse) by the i.v. route, and then ovalbumin (OVA) (Sigma-Aldrich, ref. A5503) at weeks 14, 16 and 18 (100µg in 100µL saline, emulsified in complete Freund’s adjuvant (FA) for the first injection and in incomplete FA for the subsequent ones) by the subcutaneous route. They were bled at weeks 14 (control), 17 and 19. For continuous labeling experiments with bromodeoxyuridine (BrdU) *in vivo*, mice received first an intraperitoneal injection of BrdU (1mg/20g body weight; Sigma-Aldrich, ref. B5002) and then BrdU in drinking water for 10 days (0.8mg/mL). They received a single i.v. dose of P140 5 days after they received intraperitoneal injection of BrdU. Cell subsets were analyzed by flow cytometry.

Proteinuria, dermatitis and levels of antibodies to dsDNA were recorded in MRL/lpr mice (and CBA/J mice as control) as described (25). Urine protein levels were assessed semi-quantitatively using albumin reagent strips (0 = none, 1 = 30 to 100mg/dL, 2 = 100 to 300mg/dL, 3 = 300 to 1000mg/dL, 4 = >1000mg/dL; Albustix, Miles Scientific). The titer of circulating dsDNA antibodies was defined as the inverse of the dilution giving an absorbance value of 1.0 in our ELISA conditions (25).

### Combinatorial Diversity Analysis of Mouse TRB and IGH

Throughout this study, the mouse murine T cell receptor β-chain (mTRB) VJ and immunoglobulin heavy (H) chain VJ (IGH VJ) genes are designed according to the international ImMunoGeneTics database (http://www.imgt.org). Samples were analyzed with ImmunTraCkeR kits, using the ImmunID IFS platform (ImmunID). Detailed protocols and expression of results have been published previously (41, 42). Briefly, three days after peptide (100µg per mouse) or placebo treatment, genomic DNA was extracted from CBA/J and MRL/lpr PBMCs or spleen cells. For each sample, multi-N-plex PCR was performed using an up-stream primer specific of all functional members of a given TRBV family and a downstream primer specific of a given TRBJ segment. An independent multi-N-plex PCR was also applied for IGHV and IGHJ amplification. These assays allow for the simultaneous detection of several V–J rearrangements in the same reaction. Using this technique, it was possible to detect 209 different mTRBV–TRBJ and 92 mIGHV-IGHJ rearrangements. All V–J1, J2, and Jn PCR products were separated according to their size. PCR products were separated on a 0.8%-agarose gel, directly stained with SYBR Green I, and quantified using a charge-coupled device camera equipped with a BIO-1D quantification software (Vilbert Lourmart). Constel ID software (ImmunID) was used for further analytical studies including the generation of three-dimensional repertoire illustration. Results are expressed as percentages of detected rearrangements among the total 209 mTRB and 92 mIGH possible combinatorial rearrangements. Of note, due to high number of sub-members in the V1 family, the latter one has been splitted into 8 tubes (V1a to V1h). IGH V1a comprises sub-members: V1-11, 12, 15, 67; IGH V1b: V1-49, 63; IGH V1c: V1-17-1, 18, 26, 34, 22, 62-2, 71, 76; IGH V1d: V1-14, 47, 80; IGH V1e: V1-5, 7, 4; IGH V1f: V1-20, 31, 37, 39, 42, 43, 66, 75, 77, 84, 78; IGH V1g: V1-9, 19, 36, 62-1, 58; IGH V1h: V1-50, 52, 53, 54, 55, 56, 59, 61, 62-3, 64, 69, 72, 74, 81, 82, 85. For the other IGH Vn families, they comprise all corresponding members.

### Antibodies

The following antibodies were used: for the BrdU experiments, fluorescein isothiocyanate (FITC)-labeled CD3 monoclonal antibody (mAb) clone 145-2C11, peridinin chlorophyll (PerCP)-cyanin 5.5-B220 mAb clone RA3-6B2, phycoerythrin (PE)-CD138 mAb clone 281.2; for defining the cell subtypes that repopulate the peripheral blood, PerCP-Cy5.5-CD3 mAb clone 145-2C11, allophycocyanin-B220 mAb clone RA3-6B2, FITC-Ly6G mAb clone 1A8, PE-Gr1 mAb clone RB6-8C5, allophycocyanin-CD138 mAb clone 281.2, PerCP-Cy5.5 -CD19 mAb clone 1D3; for the B cell studies, FITC-CD19 mAb clone 1D3 and allophycocyanin-CD138 mAb clone 282-2 (all from BD PharMingen). For evaluating lymphocyte viability, splenocytes were stained with PE-Cy7-CD3e (Invitrogen, ref. 25-0031-82), eFluor506-CD45R/B220 (Invitrogen, ref. 69-0452-82), Alexa Fluor (AF) 700-CD4 (Invitrogen, ref. 56-0041-82), allophycocyanin-Cy7-CD8a (Molecular Probes, ref. A15386), PerCP-Cy5-CD25 (Invitrogen, ref 45-0251-82) and FITC-FOXP3 (Invitrogen, ref. 11-4776-42) mAbs. For the comparison of leukocytes in WT and T-deficient MRL/lpr mice, staining was done with FITC-TCRαβ (Invitrogen, ref. 11-5961-82), PE-TCRγδ (Invitrogen, ref. 12-5711-82), PerCP-Cy5.5-Ly6C (Invitrogen, ref. 45-5932-82), Pe-Cy7-CD45 (Invitrogen, ref. 25-0451-82), allophycocyanin-CD8a (Invitrogen, ref. 17-0081-82), AF700-CD3 (Invitrogen, ref. 56-0032-82), allophycocyanin-Cy7-CD4 (Invitrogen, ref. A15384), eFluor506-Ly-6G/Ly-6C (Invitrogen, ref. 69-5931-82), SB600-CD25 (Invitrogen, ref. 63-0251-82), SB702-B220 (Invitrogen, ref. 67-0452-82), PerCP-Cy5.5-CD19 (Invitrogen 45-0193-82) mAbs, and LIVE/DEAD Fixable Violet stain Super Bright 436 (Invitrogen, ref. L34955). Stained cells were analyzed on an Attune NxT cytometer (Invitrogen) and FlowJo software (TreeStar).

### Viability of Cells Incubated *In Vitro* in the Presence of P140

Spleen cells of 11-13 week-old female MRL/lpr, CBA/J and C57BL/6 mice were cultured in 24-well plates (2x10^6^ cells/well) were incubated for 24h at 37°C with increasing concentrations of P140. They were stained and analyzed by flow cytometry as above.

### BrdU Intracellular Cell Staining

Intracellular cell staining was performed using the allophycocyanin-labeled BrdU flow kit (BD, ref. 552598). Briefly, 0.5x10^6^ cells per condition were stained with surface markers, washed and permeabilized using cytofix/cytoperm solution and cytoperm permeabilization buffer plus. Cells were washed and incubated with DNase (30µg per 10^6^ cells). In a last condition, cells were stained with anti-BrdU-allophycocyanin-labeled antibody, 20min at room temperature, washed and analyzed by cytometry.

### Peripheral Hypercellularity Measurement

The method used was as described previously (18, 43). The number of leukocytes/mL in the peripheral blood was evaluated by counting cells five days after a single injection of P140 peptide (100µg/mouse) into MRL/lpr or CBA/J mice. White total cells and cell subsets were analyzed by flow cytometry.

### Quantification of Serum Cytokines

For measuring IL-2, IL-4, IL-6, TNFα, IFNγ, IL-17a and IL-10 serum levels, we used the BD cytometric beads array mouse Th1, TH2, Th17 kit (BD, ref. 560485) according to the manufacturer protocol. Briefly, 25µL of MRL/lpr sera were diluted 1:1 with assay diluent, incubated with 50µL of capture beads and 50µL of Mouse Th1/Th2/Th17 PE detection reagent. Assay tubes were incubated for 2h at room temperature and protected from light. After one wash with 1 mL of wash buffer, beads were resuspended with 300µL of wash buffer and acquired on a FACSCalibur cytometer. A standard curve established with known concentrations of each cytokine was used for quantification.

### PC Differentiation and Quantification of IgM-Secreting Cells by ELISpot Assay

Splenic B cells were isolated from MRL/lpr mice by negative selection (Pan B cell isolation kit mouse; Miltenyi Biotec, ref. 130-104-443). Biotinylated anti-CD138 antibodies (1µg/mL; clone 281-2, BD Pharmingen) were added to deprive preexisting PCs. MRL/lpr B cells (97-99% purity, as estimated by flow cytometry) were then let to differentiate for 5 days in the presence of anti-CD40 antibody (0.5µg/mL; BD Pharmingen, ref. 4045566), IL-21 (10ng/mL; R&D, ref. 594-ML-010) and increasing concentrations of P140. Antibodies secretion was measured by ELISpot as previously described with minor modifications (44). Briefly, 96-well multiscreen plats (Millipore) were coated 1h at 37°C with anti-mouse IgM or IgG (1µg/mL; Jackson ImmunoResearch) in phosphate-buffered saline (PBS), pH 7.4. After washing with PBS, membranes were saturated for 1h with RPMI 1640 medium supplemented with 10% (v/v) fetal calf serum, 0.05mM β-mercaptoethanol, 10µg/mL gentamycin and 10 mM HEPES. Serial dilutions of differentiated B cell suspensions were incubated for 18-24h at 37°C. The last steps were as described (18) using anti-mouse IgM (µ chain-specific) biotin-labeled Ig or anti-mouse IgG (Fcγ-specific) biotin-labeled Ig (both 1:20,000 in PBS containing 0.05% (v/v) Tween (PBS-T); Jackson ImmunoResearch) that were added for 12h at 4°C, followed by alkaline phosphatase-labeled extravidin (Sigma-Aldrich) for 1h at 37°C and 5-bromo-4-chloro-indolyl phosphate and nitro-blue tetrazolium chloride substrate (Sigma-Aldrich). The reaction was stopped with water when spots were clearly visible. Spots were counted with a Bioreader4000 (BioSysGmbH). The results were expressed as the number of IgM or IgG-secreting cells/10^6^ total cells using the following formula: (number of spots/well – background measured in control wells incubated with PBS or BSA) x dilution factor of the cell suspension.

### Ethics Statement

Animal protocols were carried out with the approval of the local Institutional Animal Care and Use Committee (CREMEAS, Strasbourg, France) and the French Ministère de l’Enseignement Supérieur de la recherche et de l’innovation (APAFiS#2016112916066575 and APAFiS#23210-2019120617587704). According to our agreement, and taking into account the best European practices in the field, we took the necessary measures to avoid pain and minimize the distress and useless suffering of mice during the time of experiment and killing process.

### Statistical Analysis

Statistical analyses were performed using Wilcoxon Rank Sum tests (comparison of diversity between two groups), nonparametric Mann-Whitney test, paired or unpaired t-tests when sample distribution followed a Gaussian distribution. *P* value <0.05 were considered statistically significant.

## Results

### Repopulation of Circulating Immune Cells Following P140 Treatment

**Figure 1:**
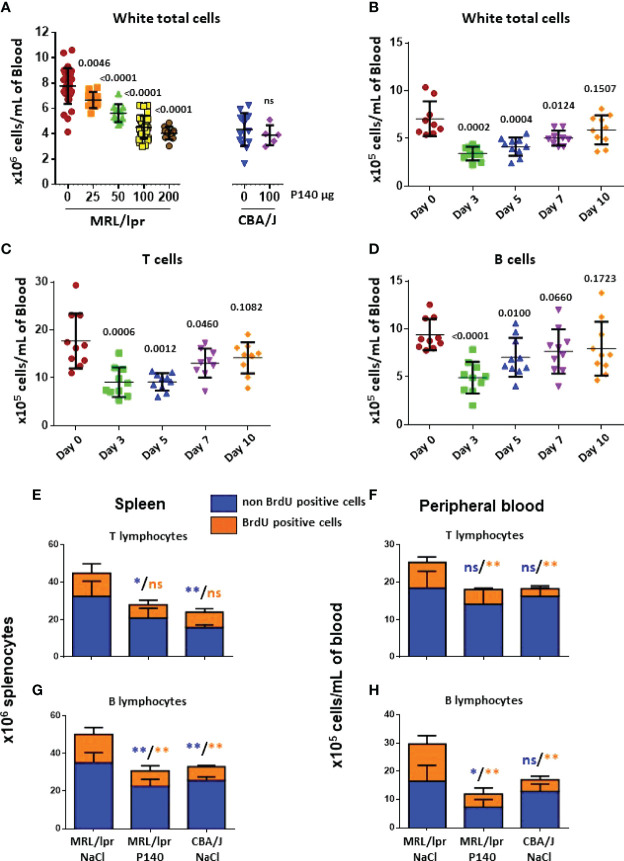
*In vivo* effect of P140 peptide on the activity of immune cells in the spleen and peripheral blood of MRL/lpr mice. **(A–D)**. Reconstitution post-P140 treatment of the peripheral immune cell pool of normal and MRL/lpr lupus mice. 11-13 week-old female CBA/J or MRL/lpr mice received a single i.v. administration of either peptide P140 in saline (increasing doses in panel **(A)**; fixed dose of 100 µg peptide/mouse in panels **(B–D)** or saline only. The number of leukocytes/mL was evaluated by counting cells 5 days later panel **(A)** or sequentially until day 10 post-P140 treatment by flow cytometry after labelling cells with appropriate fluorophores in panels **(B–D)**. Each symbol represents one individual mouse (n = 10-48 MRL/lpr mice and 5-14 CBA/J in panel **(A)**; n = 10 in panels **(B–D)**. The results mixed from 2 independent experiments are presented. The horizontal bars represent the respective average cell count values. Statistical significance was assessed using unpaired t-test. Additional data are presented in the supplementary material. **(E–H)**. Long-term *in vivo* BrdU incorporation in various immune cell subsets of CBA/J and MRL/lpr mice that received or not P140 peptide. Age-matched mice received each a first intraperitoneal injection of BrdU, which was then administrated in drinking water for 10 days. 10-11 week-old female MRL/lpr mice treated with P140 peptide were given a single i.v. dose of peptide 5 days after they received intraperitoneal injection of BrdU. Cells were collected from the spleen and PBMCs, stained by incubating them with fluorescently-tagged antibodies to appropriate surface markers, fixed stained with anti-BrdU antibodies, and analyzed by flow cytometry. Histograms represent the mean ± SD of BrdU ^+^ (cycling) or ^–^ (non-cycling) cells derived from 5 mice per group. Statistical significance was assessed using the Mann Whitney test. *p < 0.05; **p < 0.01; ns, non-significant. Additional data are presented in the supplementary material.

We previously showed that P140 administration into MRL/lpr mice causes egress of several immune cell subtypes from the peripheral blood of treated mice without affecting T cell priming and the ability of P140-teated mice to resist to an infectious viral challenge (18, 22, 23, 39). This effect was very specific and was not seen with the numerous P140 analogues that were tested (18). To analyze further the characteristics of remaining cells, we first studied this egress from a dynamic viewpoint. The depleting effect of P140 was peptide dose-dependent until a threshold dose of 100µg P140/mouse/injection, which led to a peripheral cell balance similar to the one measured in healthy mice (**Figure 1A**). Increasing P140 dose at 200µg P140/mouse did not affect this balance further. Using the optimal dose of 100µg/mouse, we found that at day 3 post-P140 treatment, there was a drop of T cell (including DN T cell), B cell, PC, monocyte and granulocyte counts (**Figures 1B–D** **;** **Supplementary** **Figure 1**). On day 10 post-P140 treatment, the counts of monocytes and granulocytes still remained low, in contrast to the counts of T cells, DN T cells, B cells and PCs that reached again the basal level measured in untreated MRL/lpr mice.

### Effects *In Vivo* of P140 on Proliferative Activities of MRL/lpr Immune Cells

To investigate the functional status of T and B cells in the “depleted” compartment, then we measured the incorporation of BrdU in various immune cell subsets of CBA/J and MRL/lpr mice that received or not P140. Three groups of five mice each, were tested, namely MRL/lpr mice that received NaCl only (group 1), MRL/lpr mice that received P140 in saline (group 2) and control CBA/J mice that received NaCl only (group 3). Mice received a first i.v. injection of BrdU and then permanently, in drinking water, for 10 days. P140 was given once, intravenously, on day 5. On day 10, flow cytometry analysis of cell subtypes showed that in the spleen, although there were fewer T cells in group 2 compared to group 1, there was no change in the number of dividing T cells (BrdU^high^) (**Figure 1E**). As expected the number of cycling cells was elevated in the MRL/lpr DN T cell subset with regard to normal mice but these numbers were not different in treated and non-treated animals (**Supplementary** **Figure 2A**). In the PBMC fraction, in contrast, the total number of non-dividing (long-lived) T cells was not different in the three groups (**Figure 1F**), while the number of BrdU^high^ T cells, which was raised in MRL/lpr mice compared to normal mice, was significantly reduced upon P140 treatment (**Figure 1F**). The proliferation of blood circulating DN T cells was apparently not modified upon P140 treatment (**Supplementary** **Figure 2B**).

**Figure 2:**
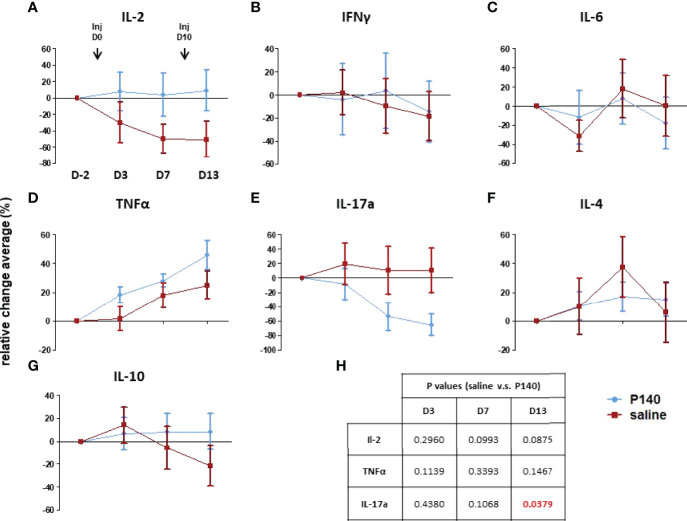
P140 alters the serum cytokine profile. **(A–G)** Two groups of MRL/lpr mice were injected i.v. with either 100µg/100µL of P140 (n = 16) or NaCl only (n = 20) on day 0 and 10. Sera were collected on day -2, 3, 7 and 13 and tested individually for their content in IL-2, IL-4, IL-6, IL-10, IL-17a, INF-γ and TNF-α. Due to substantial inter-mouse variability, cytokine secretion was measured in individual MRL/lpr mice and normalized, and the results were expressed in terms of cytokine levels relative changes (± SEM) in each mouse, overtime, in comparison to day -2. **(H).** Statistics. A paired t-test was used to compare the data obtained in each group on day 3, 7 and 13 relative to day -2. An unpaired t-test was applied to compare the two groups at each time point.

Regarding the B cell compartment, BrdU pulse chase experiments showed that the number of dividing and non-dividing MRL/lpr B cells was decreased upon P140 treatment both in the spleen and PBMC fraction (**Figures 1G, H** **)**. Although no change was found in the spleen, fewer dividing PBs and PCs were counted in the peripheral blood (**Supplementary Figures 2C– F**). It has to be noticed however that although these results are statistically significant, they should be approached with cautious since the number of PCs and PBs was very small.

Together, these analyses indicate that following a single i.v. administration of P140, the proliferative status of total MRL/lpr T cells evaluated 5 days later is globally not changed in the secondary lymphoid tissue (spleen), while it is affected in the peripheral blood compartment. In parallel, the number of proliferating B cells is reduced both in the spleen and peripheral blood compartments. The proliferating rate of PBs and PCs, which remains broadly stable upon P140 administration tended to diminish in the peripheral blood.

### Effect of P140 Treatment on the Pattern and Levels of Circulating Cytokines

The above data suggest that post-P140 treatment (one single i.v. injection), there is a window of about 10 days during which immune cell abnormalities seem to be reduced in the peripheral blood of MRL/lpr mice. Over this period, we found that while the level of blood-circulating IL-2, IL-4, IL-6, IL-10 and IFN-γ showed no or marginal variations in treated *vs*. untreated mice (**Figure 2**), there was a sharp significant drop of soluble IL-17a levels in P140-treated mice. Type-I interferon levels were not tested here since in contrast to other mouse models of lupus, MRL/lpr mice do not show evidence of type-I IFN signature (45).

These result, together with previous data showing that upon treatment with P140, a number of cytokine-encoding genes are significantly up- or down-regulated in non-activated lymph node CD4^+^ T cells (23), underline that the profile of secreted cytokines is shifted upon P140 treatment.

### TCR Repertoire of Blood Circulating Cells in P140-Treated and Non-Treated MRL/lpr Mice

To gain relevant information regarding the characteristics of circulating lymphocytes that remain in the blood of mice shortly after P140 administration (“depleting” phase), next we analyzed the respective TCR and BCR repertoire of circulating T and B cells. The genomic DNA was prepared from PBMCs and spleen cells, 3 days post-P140 treatment, i.e. within the time window of P140 effect identified above at the periphery. Because of lack of documented information in the existing literature, the first step of this investigation was to compare TCR and BCR V-J rearrangements in CBA/J and MRL/lpr mice, which share the same MHC and mTRB haplotypes (46), and thereafter, the possible influence of P140 on these rearrangements. The experiments and the results are described in full detail in the supplementary material. The most relevant results are summarized below.

**Figure 3:**
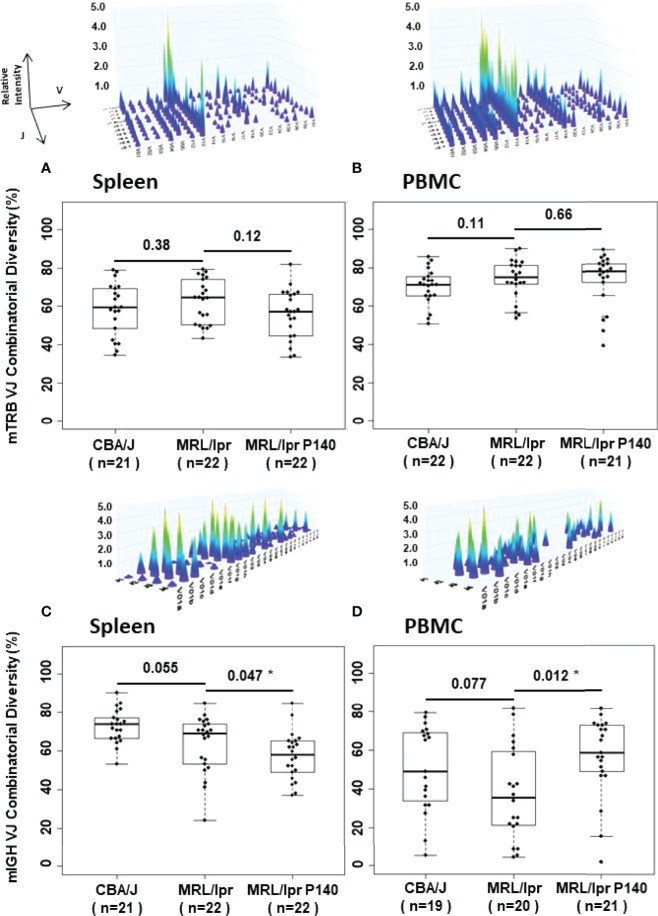
Dot, box plot and 3-D graph representations of combinatorial mTRB and mIGH diversity distribution in spleen and PBMC samples from CBA/J and MRL/lpr mice. The 4 representative images illustrate 3-D graph of the TRB and IGH immune repertoires generated by Constel ID software (ImmunID). Each peak represents the rearrangement of a VH (or VB) gene family relative to the rearrangement of JH (or JB) genes. Relative intensity of these rearrangements is represented on the z-axis. Graphs have been generated for each dot in **(A–D)**. Combinatorial diversity (%) median into box plots are represented by the central line. n = number of samples examined. Comparison of diversity by Wilcoxon Rank Sum tests between two groups is indicated. *p < 0.05.

A thorough analysis of mTRB and mIGH diversity and combinatorial repertoire composition was performed from spleen and PBMC samples of CBA/J and MRL/lpr mice. We used a general immune companion diagnostic assay to monitor T and B cell responses and evaluate the immune status in the different strains of mice (41). The results were compared for each group of mice and for each repertoire. A total of 43 spleen and 44 PBMC samples were analyzed for mTRB repertoire, and 43 spleen and 39 PBMC samples were analyzed for mIGH repertoire (**Supplementary Table 1**). When CBA/J and MRL/lpr mice samples were compared, no statistically significant difference was observed in terms of mTRB and mIGH combinatorial diversity (**Figure 3** **;** **Supplementary Figures 3A, B, D, E**). However, the analyses of VJ rearrangements showed the existence of some specific mTRB VJ rearrangements that were statistically more frequently represented in MRL/lpr mice compared to CBA/J mice and could therefore be designed as a signature. No notable mIGH VJ repertoire signature was revealed (For full details see Supplementary material;**Supplementary Figures 4, 5** **;** **Supplementary Tables 1–3**).

Next, we examined the influence of P140 on these rearrangements. When untreated and P140-treated MRL/lpr mice were compared, no difference was observed in splenocytes and PBMCs in terms of mTRB VJ combinatorial diversity (**Figures 3A, B** **;** **Supplementary Figures 3B, C** **)**. The analysis done on the entire mTRB VJ repertoire (**Supplementary** **Figures 4**) revealed that compared to untreated MRL/lpr mice, one mTRB VJ rearrangement only in spleen and none in PBMCs was significantly more frequent in P140-treated mice (**Supplementary Figures 6A** **;** **Supplementary Table 4**). However, mTRB VJ rearrangements were found more frequently in untreated MRL/lpr mice compared to P140-treated mice. From those mTRB VJ rearrangements, V29-J2.1 and V3-J2.3, which were found both in splenocytes and PBMCs from untreated mice, could represent a signature that is absent in P140-treated mice. Regarding the fate of the mTRB VJ rearrangements distinguishing MRL/lpr spleen from CBA/J mice, some (V3-J2.3, V26-J2.4, V29-J2.1) also present a significant difference of frequency between treated and untreated MRL/lpr mice, meaning that they are apparently affected by the treatment. The analysis done with PBMC samples led to the same conclusions, which concern mTRB VJ rearrangements V29-J2.5 and V29-J1.5. These findings collectively suggest that pre- and post-P140 treatment, the distribution of frequencies of some mTRB VJ rearrangements is changed. To reinforce this assumption, we therefore compared the repertoire composition of the P140-treated MRL/lpr mice *versus* CBA/J control mice (**Supplementary Figure 7** **;** **Supplementary Table 5**). While P140 seemed to have no influence on the representation of certain rearrangements (see the details in the supplementary material), the frequency of others (V3-J2.3, V26-J2.4, and V29-J2.1 in spleen and V29-J2.5 and V29-J1.5 in PBMCs) were found levels that were very similar in P140-treated mice and CBA/J mice (no significant difference of frequency could be highlighted for these rearrangements between the two groups; **Supplementary Figure 7A**). These results highlight that treatment of MRL/lpr mice with P140 has an impact on the distribution frequency of rare mTRB VJ rearrangements observed in MRL/lpr prior to treatment, which reach levels similar to those found in healthy CBA/J mice.

**Figure 4:**
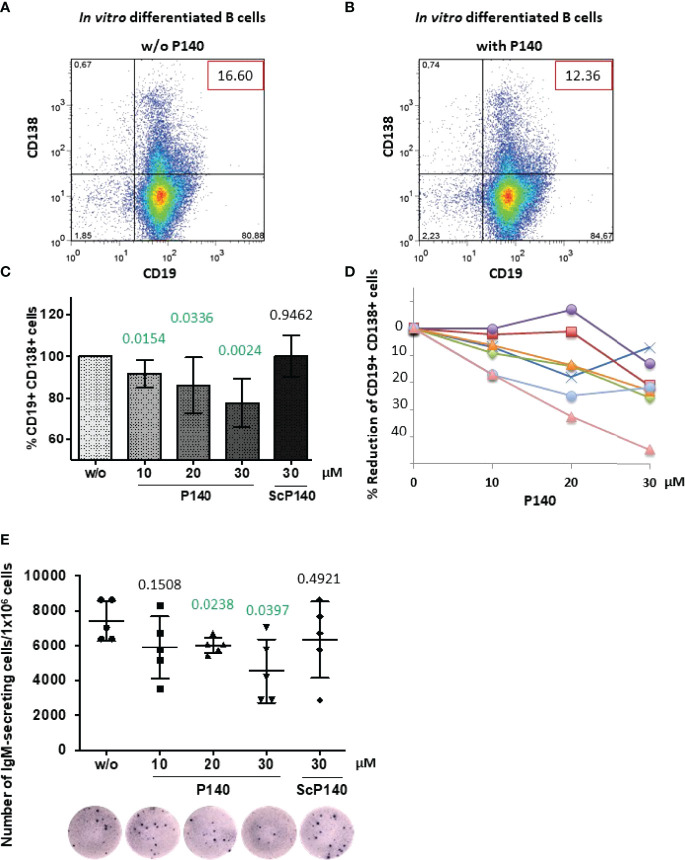
P140 peptide alters the ability of MRL/lpr B cells to differentiate. B cells collected from 12-13 week-old female MRL/lpr mice were let to differentiate *in vitro* in the presence of anti-CD40 antibodies and IL-21 and in the presence (with) or absence (w/o) of P140 peptide added at a 10, 20 and 30-µM concentration. The scrambled peptide ScP140 was used as control. Differentiation was assessed 5 days later by flow cytometry by evaluating CD19 and CD138 expression. We verified that apoptosis did not occur until at least 6 days after adding P140 or ScP140 in the culture. **(A, B)** A dot-plot of each condition is shown (representative of 7 independent experiments). The percentages of B cells expressing or not CD19 and CD138 are gathered in the graph. **(C, D)** The mean percentages of CD19^+^CD138^+^ PBs ± SD in panel C (n = 7) and reduction percentages illustrated in the case of 7 individual mice [panel **(D)**] are shown as a function of P140 concentration. Statistical significance was assessed using unpaired t-test. **(E)** The frequency of IgM-secreting cells was measured by ELISpot. The results are expressed as the number of IgM-secreting cells/1x10^6^ total cells. A photograph of one culture well is shown for each condition (representative of 5 independent experiments corresponding to 5 individual mice). Statistical significance was assessed using Mann-Whitney test.

With regard to mIGH VJ repertoires of MRL/lpr mice treated or not with P140 (**Supplementary** **Figures 4C, D** **)** the comparison revealed that more frequent differences occur in PBMCs than in spleens, where 22 rearrangements were found significantly more often in P140-treated than in untreated MRL/lpr mice (**Supplementary Figure 6B** **;** **Supplementary Table 4B**). As also found in the case of mTRB VJ rearrangements, the distribution of the frequency of mIGH VJ rearrangements was affected by P140 treatment. However, since in our hands no mIGH VJ repertoire signature could be visualized in MRL/lpr B cells, it was not possible to investigate a potential effect of peptide on this specific repertoire with regard to the one of healthy mice (**see details in** **Supplementary Figure 7B** **;** **Supplementary Table 5B**).

### Effects of P140 on the Capacity of MRL/lpr B Cell to Differentiate *In Vitro* Into Ig-Secreting PCs

To further decipher the downstream aftermath of P140, next we looked at whether P140 could modify the capacity of MRL/lpr B cells to differentiate into CD138/Syndecan-1^+^ PBs and PCs. We addressed this question *in vitro* using purified B cells from 12-week-old MRL/lpr mice, which were let to differentiate for 5 days in the presence of anti-CD40 antibody and IL-21 (47, 48). The number of CD19^-^CD138^+^ PCs was too low in the cultures to support any reliable conclusion (**Figures 4A, B**). However, we observed that the percentage of CD19^+^CD138^+^ PBs diminished in a P140 concentration-dependent manner (**Figures 4C, D**). A scrambled control analogue (ScP140) had no measurable effect. These results corroborate our previous flow cytometry results (23) showing the effect of P140 on CD138^+^B220^-^ PCs, measured 20h after MRL/lpr PBMCs treatment, and data generated with cells from lupus patients treated *ex vivo* with P140 and ScP140 (40).

Since the size of CD19^+^CD138^+^ PBs compartment was significantly reduced in the presence of P140, we assessed the effect on Ig production and the number of antibody-secreting cells. The latter, which represent the terminally differentiated cells of the humoral immune response, are the main effector B cells that expand in acute SLE (49). Of note, autophagy has been seen to play a general role in the late stages of B cell activation and subsequent PC differentiation (50–52). In the experimental *in vitro* setting we applied, naïve B cells were not able to efficiently class-switch and therefore the fine specificity of secreted Ig could not be identified. However, ELISpot results clearly indicated that in a P140 concentration-dependent manner, the frequency of IgM-secreting cells was significantly diminished in the culture (**Figure 4E**). ScP140 had no measurable effect.

### P140-Treated MRL/lpr Mice Respond Normally to an Exogenous Immunogen

**Figure 5:**
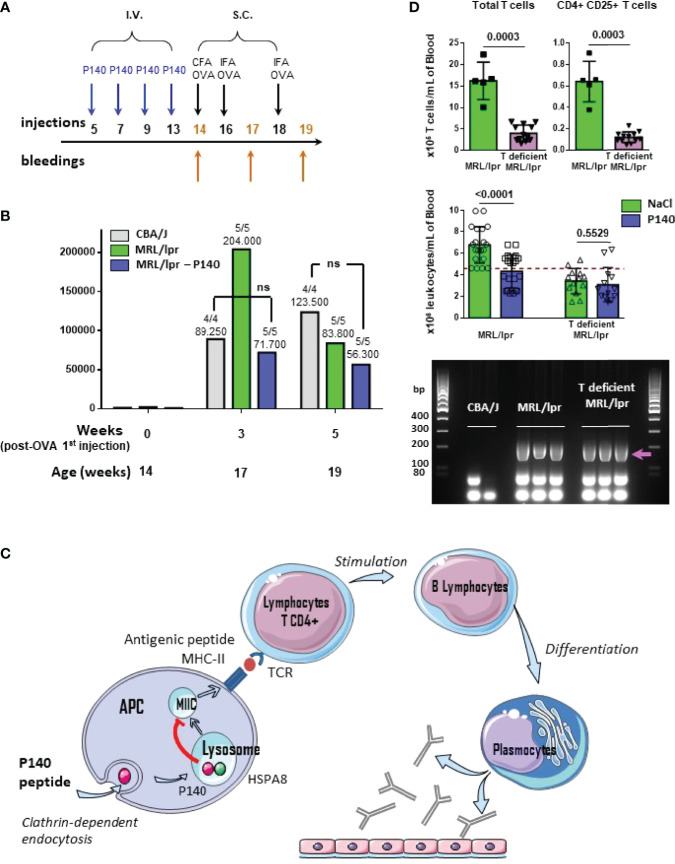
P140 mechanism of action. **(A, B)** P140 does not affect the ability of treated mice to mount an efficient response elicited by a foreign model immunogen. **(A)** Protocol of injections and bleedings used in this experiment. **(B)** The sera collected from CBA/J, untreated and P140-treated MRL/lpr mice that received ovalbumin (OVA) as model foreign immunogen, were tested by ELISA for their capacity to produced anti-OVA IgG antibodies. The trend shown here was confirmed in two independent experiments that altogether involved a total of 15 mice/study group (one of two independent experiments is shown). The titers were defined as the reciprocal of the dilution giving an absorbance value of 0.5. The average titer and the number of responder animal (titer>250) are indicated at the top of each histogram. P values measured using Mann-Whitney test. ns, non-significant. **(C)** Schematic representation of the domino mechanism of action of P140. **(D)** Characteristics of 13 week-old T-deficient MRL/lpr mice and comparative effect of P140 in 13-week old T-deficient and WT MRL/lpr mice. Top: number of total peripheral T cells and activated CD4^+^CD25^+^ cells per mL in 5 WT MRL/lpr and 12 T-deficient MRL/lpr mice; P values measured using Mann-Whitney test. Middle: number of white blood cells per mL, before and after P140 treatment (n = 20 and 13 in the group of WT MRL/lpr and T-deficient MRL/lpr mice, respectively); P values measured using unpaired t test. Bottom: genotyping of *Fas* as determined by PCR of genomic DNA from the tail of mice (n = 2-3 mice/group taken randomly). CBA/J mice were used a negative control. The arrow indicates the position of the band to detect *Fas.* Additional data are presented in the **Supplementary Material**.

Considering the changes of immune cell distribution and phenotypes described above, at this stage it was important to ensure that P140-treated mice were still able to mount a classical response to a foreign antigen. We immunized untreated and P140-treated MRL/lpr mice with soluble OVA used as a model immunogen and evaluated the antibody response by ELISA (**Figure 5A**). A strong anti-OVA IgG antibody response was measured in all mice of each study group. Compared to healthy mice, the mean anti-OVA antibody titers were particularly elevated in MRL/lpr mice after the second administration of immunogen, and then tend to decrease (**Figure 5B**). This hyper-responsiveness was not seen in MRL/lpr that received P140 prior immunization. With the exception of this feature, the mean anti-OVA antibody titers were of the same order in immunized untreated and P140-treated MRL/lpr mice. The data corroborate our previous observations indicating that P140 treatment did not affect the capacity of MRL/lpr mice challenged with an infectious dose of influenza virus given intranasally, to mount specific T- and B-cell responses against viral antigens and to recover from infection (39). Taken together, these past and present data convincingly support that the immune cells and associated molecular mechanisms are competent following administration of P140 to mice and able to develop an appropriate immune response to a given antigen. This indicates that P140 does not behave as an immunosuppressant and instead would help restore immune homeostasis.

### P140 Exerts No Effect in the Absence of Functional T Cells

Previous data have shown that *in vivo*, P140 has no direct effect on post-BCR signaling of memory, naïve mature, transitional and B1 cells (40). Among other outcomes (22, 24, 40), these findings led us to propose that the effect of P140 likely occurs *via* a mechanism involving upstream steps of T cell regulation (T cell activation, proliferation and ability of signaling) with a strong downstream impact on B cell maturation and differentiation into antibody-secreting cells (**Figure 5C**). This scheme is further comforted here by our findings that *in vitro*, P140 has no direct impact on precursors (CD3^+^, B220^-^, CD4^+^, CD8^-^, CD25^-^ , FOXP3^+^) and mature (CD3^+^, B220^-^, CD4^+^, CD8^-^, CD25^+^, FOXP3^+^) Tregs from MRL/lpr and healthy mice of different haplotypes (**Supplementary Figure 8**).

In this study, we confirm even further the sequence of events of the P140’ mechanism of action in showing that in the absence of activated peripheral CD4^+^ T cells, P140 exerts no protecting effect in MRL/lpr mice (**Figure 5D** **;** **Supplementary Figures 9, 10** **).** We unexpectedly identified a phenotypic drift of our in-house colony, which was a progeny of mice obtained from the Jackson Laboratory (MRL/MpJ-Faslpr/J mice; stocking number #00485). These mice displayed both cellular and clinical differences compared to wild-type (WT) MRL/lpr mice. While they retained expected genotype of WT MRL/lpr mice, as attested by classical genotyping of *Fas* gene by PCR (**Figure 5D**), and several pathophysiological characteristics of the WT MRL/lpr strain (splenomegaly, enlarged lymph nodes and salivary glands; **Supplementary Figures 9A–C**), they strikingly present a milder disease with a reduced level of proteinuria (**Supplementary Figure 9D**) and a decreased count of peripheral cells compared to MRL/lpr mice (**Figure 5D**; **Supplementary Figure 10**). The levels of circulating IgG antibodies to ds DNA were elevated in both, WT and T-cell defective MRL/lpr mice (titers were ~100, 1680 and 2145 in CBA/J, WT and T-deficient MRL/lpr mice, respectively, as measured by ELISA and calculated at an absorbance value of 1.0 in ELISA). We noted that in contrast to WT MRL/lpr mice, which often have a few (1, 2) successive litters of pups only, T-cell defective MRL/lpr mice had greater number of births (a full description of these mice will be published elsewhere). Detailed study of peripheral immune cell subsets showed that the myeloid lineage (granulocytes and monocytes) remained similar in the WT and spontaneously-derived MRL/lpr mice. However, in the latter the lymphoid lineage cells were significantly affected in the peripheral blood (**Supplementary Figure 10**). The number of circulating CD4^+^, CD8^+^, and DN T cells was significantly lower compared to WT MRL/lpr mice. Overall, the number of activated CD25^+^CD4^+^ T cells that are characteristic of the WT MRL/lpr mouse, was greatly reduced, and found at the baseline observed in healthy CBA/J mice (**Figure 5D**; **Supplementary Figure 10** **)**. The compartment of unconventional T cells expressing a γδ TCR (a relatively small subset of T cells in peripheral blood that can be activated by lipids and phosphoantigens) was affected just like T cells expressing TCR α- and β-chains. An important result is that T-cell deficient MRL/lpr mice appeared totally refractory to P140 treatment (**Figure 5D**), which reinforces our operational scheme (**Figure 5C**). This finding is consistent with previous results generated in WT MRL/lpr mice depleted in γδ T cells (23). It corroborates past results showing the effects resulting from the depletion of cells of the lymphoid lineage on the course of the disease in WT MRL/lpr mice (38, 53, 54).

## Discussion

The present study sought to dissect the molecular basis of the therapeutic effect of peptide P140 given to patients for SLE indication (19, 20). Our past investigations led us to characterize the upstream mechanism of action of P140 when the peptide enters cells and homes into lysosomes of MRL/lpr mice, where it compromises CMA that is abnormally upregulated in MRL/lpr mice (24, 26). We demonstrated that P140 induced a decreased expression of excessive MHCII molecules in mouse and human APCs (22, 25, 40) and a weaker or no priming of autoreactive MRL/lpr CD4^+^ T cells (39). These results could easily be explained by the binding of P140 to HSPA8 (18, 22), its subsequent destabilizing effect on the HSPA8/HSP90AA1 chaperone complex (22) and LAMP2A expression (26) [hence on CMA (24, 26)], which are crucial in the MHCII presentation of antigens to CD4^+^ T cells (33–35, 37). This domino process has a beneficial downstream result in lupus. Indeed, if autoreactive CD4^+^ T cells are no longer activated, they in turn cannot signal and activate autoreactive B cells, thus restricting their proliferation and differentiation, leading to a significant reduction in autoantibodies, especially antibodies to native DNA and an improvement in the clinical signs of the disease [as demonstrated in both mice and lupus patients (17, 19, 40)]. The unexpected generation of T-cell deficient MRL/lpr mice helped to consolidate this mechanism as these deficient mice, which had lower levels of proteinuria, reduced peripheral hypercellularity and a slightly longer half-life compared to MRL/lpr WT mice, are refractory to P140 treatment.

Overall, the results reported in the present study fully support and reinforce the overall model we proposed (19). Following the administration of P140, there is a significant decrease of activated cells that are over-represented in the spleen and peripheral blood and a decrease level of pro-inflammatory IL-17a in the serum of treated MRL/lpr mice, a cytokine involved in the pathogenesis of SLE (13). While the levels of serum autoantibodies were decreased in P140-treated MRL/lpr mice (17, 23, 25), the latter develop a classical and expected antibody response to a foreign antigen [shown here with OVA and previously against Influenza virus (39)].

One of the most important results presented in this report is that after P140 treatment, the mice retain the overall combinatorial diversity of TRB but no longer possess the combinations of TRB rearrangements observed in MRL/lpr and, instead, exhibit combinations of rearrangements of TRB similar to those identified in non-autoimmune MHC-matched mice. Since as mentioned above, P140-treated mice presented with a decreased autoantibody response but an apparent normal antibody response to foreign antigens, we propose that peptide P140 acts by depleting, at least in part, the pool of autoreactive T cell clones. This impoverishment of the harmful autoreactive T cell compartment (by T cell anergy or by depletion) may lead to normalize immune responses. P140-treated animals appear with an overall T cell repertoire that is close to the normal one in otherwise autoimmune MRL/lpr mice.

It is worth noting that the present data were obtained in a mouse model that is particularly severe in comparison with the pathophysiological features met in human SLE. The beneficial effect we describe above occurs transiently in diseased MRL/lpr mice, during a window of time following P140 administration given once here. It may be amplified following several administrations of P140, explaining the significantly longer half-life of P140-treated MRL/lpr mice that repetitively received the peptide (17, 18) and the efficacy in patients (19).

In this refined model, the place of the endolysosomal cellular axis and CMA remains a central node. This pathway is abnormally activated in lupus and very likely involved in the presentation of self-antigens to autoreactive CD4^+^ T cells. P140 could either eliminate B cell APCs that are CMA-hyper-responsive (22) or regulate their antigen-presenting functions (24). We have shown recently that P140 targets lysosomes that are CMA-positive and regulates their hyperactivity at the step of CMA substrate lysosomal uptake (26). P140 apparently does not affect the processing of foreign antigens. While counteracting the inflammatory process and regulating lysosomal alterations, P140 helps to restore physiological and antigen-presenting conditions, thereby preventing the activation of autoreactive T and B cells (55, 56).

In summary, our studies highlight an immunological explanation for the clinical benefit of P140 in MRL/lpr mice and potentially also in patients with lupus. By down-regulating the autophagic system involved in the processing of self-antigens by APC B cells, P140 is effective in depleting and eliminating autoreactive T and B cells from the immune system and restoring “normal” immune system function. This mechanism of clearance of autoreactive lymphocytes compromising the integrity of the immune system is not immunosuppressive as the immune responses observed in different models are normal. These results are therefore the more worthy of our attention. They could have many applications in immune diseases in which autophagy processes are unbalanced.

## Data Availability Statement

The original contributions presented in the study are included in the article/**Supplementary Material**. Further inquiries can be directed to the corresponding author.

## Ethics Statement

The animal study was reviewed and approved by CREMEAS and the French Ministère de l’Enseignement supérieur de la recherche et de l’innovation.

## Author Contributions

NS, LT, and MW performed the research, analyzed the data and wrote parts of the paper. EJM analyzed TCR and BCR rearrangements data. SM designed the study and wrote the paper.

## Funding

This research was funded in part by the French Centre National de la Recherche Scientifique (CNRS); the University of Strasbourg Institute for Advanced Study (USIAS); the ITI 2021-2028 program, University of Strasbourg-CNRS-Inserm, IdEx Unistra (ANR-10-IDEX-0002) and SFRI (STRAT’US project, ANR-20-SFRI-0012); the European Regional Development Fund of the European Union in the context of the INTERREG V Upper Rhine program; the FHU ARRIMAGE and the OMAGE project granted by Region Grand-Est of France and FEDER.

## Conflict of Interest

SM is named as co-inventor on CNRS-ImmuPharma patents relating to the P140 peptide. 

The remaining authors declare that the research was conducted in the absence of any commercial or financial relationships that could be construed as a potential conflict of interest.

## Publisher’s Note

All claims expressed in this article are solely those of the authors and do not necessarily represent those of their affiliated organizations, or those of the publisher, the editors and the reviewers. Any product that may be evaluated in this article, or claim that may be made by its manufacturer, is not guaranteed or endorsed by the publisher.

## Abbreviations

AF, Alexa Fluor; APC, antigen-presenting cells; BCR, B cell receptor; BrdU, bromodeoxyuridine/5-bromo-2’-deoxyuridine; CMA, chaperone-mediated autophagy; ELISA, enzyme-linked immunosorbent assay; ELISpot, enzyme-linked immunospot; FITC, fluorescein isothiocyanate; FOXP3, forkhead box P3; HSP, heat-shock protein; Ig, immunoglobulin; IL, interleukin; i.v., intravenous; LAMP2A, lysosomal-associated membrane protein; mAb, monoclonal antibody; MAP1LC3B, microtubule-associated proteins 1A/1B light chain 3B; MHC, major histocompatibility complex; MRL/lpr, Murphy Roths large/lymphoproliferation; mRNA, messenger RNA; mTRB, murine T cell receptor β-chain; OVA, ovalbumin; PB, plasma blast; PBMC, peripheral blood mononuclear cell; PC, plasma cell; PCR, polymerase chain reaction; PE, phytoerythrin; PerCP, peridinin chlorophyll; Sc, scrambled; SD, standard deviation; SEM, standard error of the mean; SLE, systemic lupus erythematosus; SQSTM1/p62, sequestosome 1; TCR, T cell receptor; TNF-α, tumor necrosis factor-alpha.
