# Supplementary material for: P140 Peptide Leads to Clearance of Autoreactive Lymphocytes and Normalizes Immune Response in Lupus-Prone Mice
Source: Front Immunol. 2022 Jun 1;13:904669. doi: 10.3389/fimmu.2022.904669 (PMC9199391; doi:10.3389/fimmu.2022.904669)
Supplement: Supplementary file 1 [file DataSheet_1.pdf]

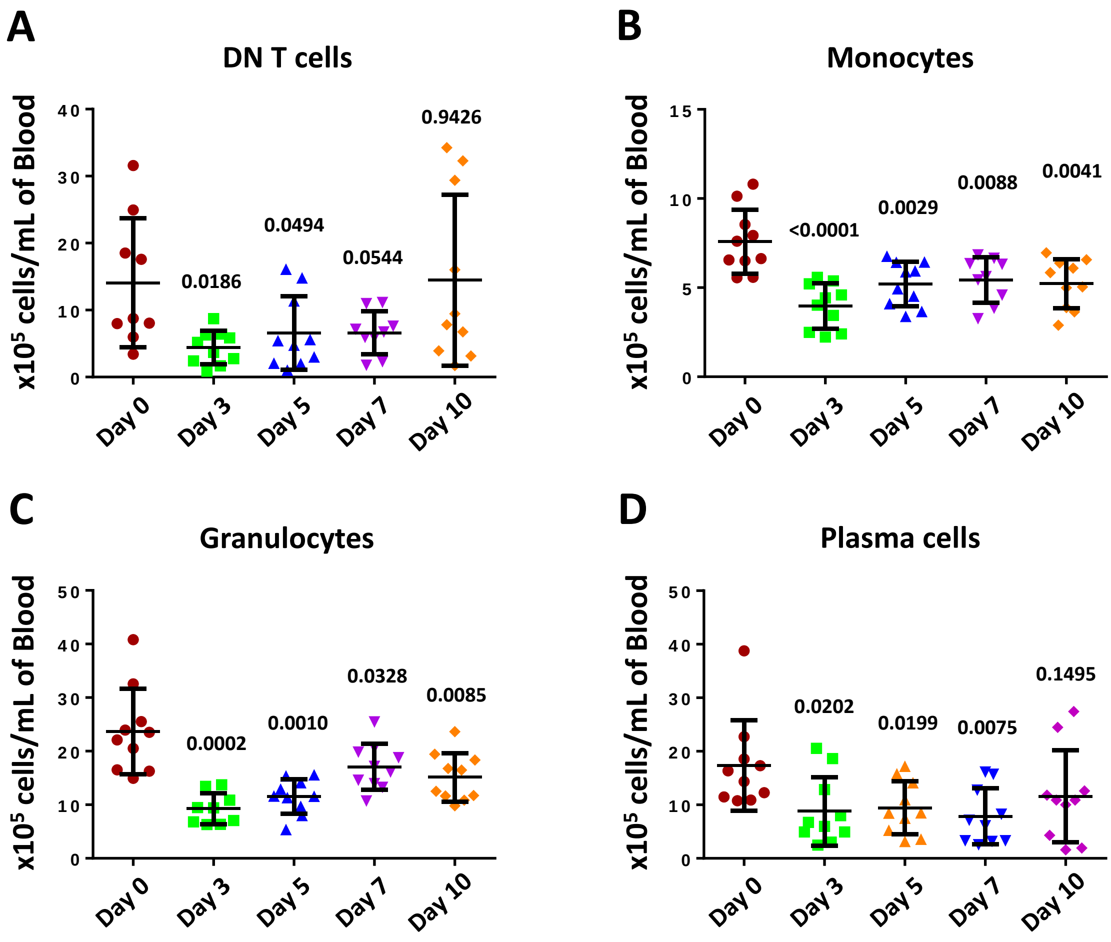

Supplementary Figure 1

Reconstitution post-P140 treatment of the peripheral immune cell pool of MRL/lpr lupus mice. 11-13 week-old female MRL/lpr mice (10 per group; a total of 50 MRL/lpr mice) received a single i.v. administration of 100 µg P140/mouse in saline. The number of cells/mL was evaluated at days 0, 3, 5, 7 and 10 post-P140 treatment, by flow cytometry, after labelling cells with appropriate fluorophores. Each symbol represents one individual mouse. The results mixed from 2 independent experiments are presented. The horizontal bars represent the respective average cell count values. Statistical significance was assessed using unpaired t-test.

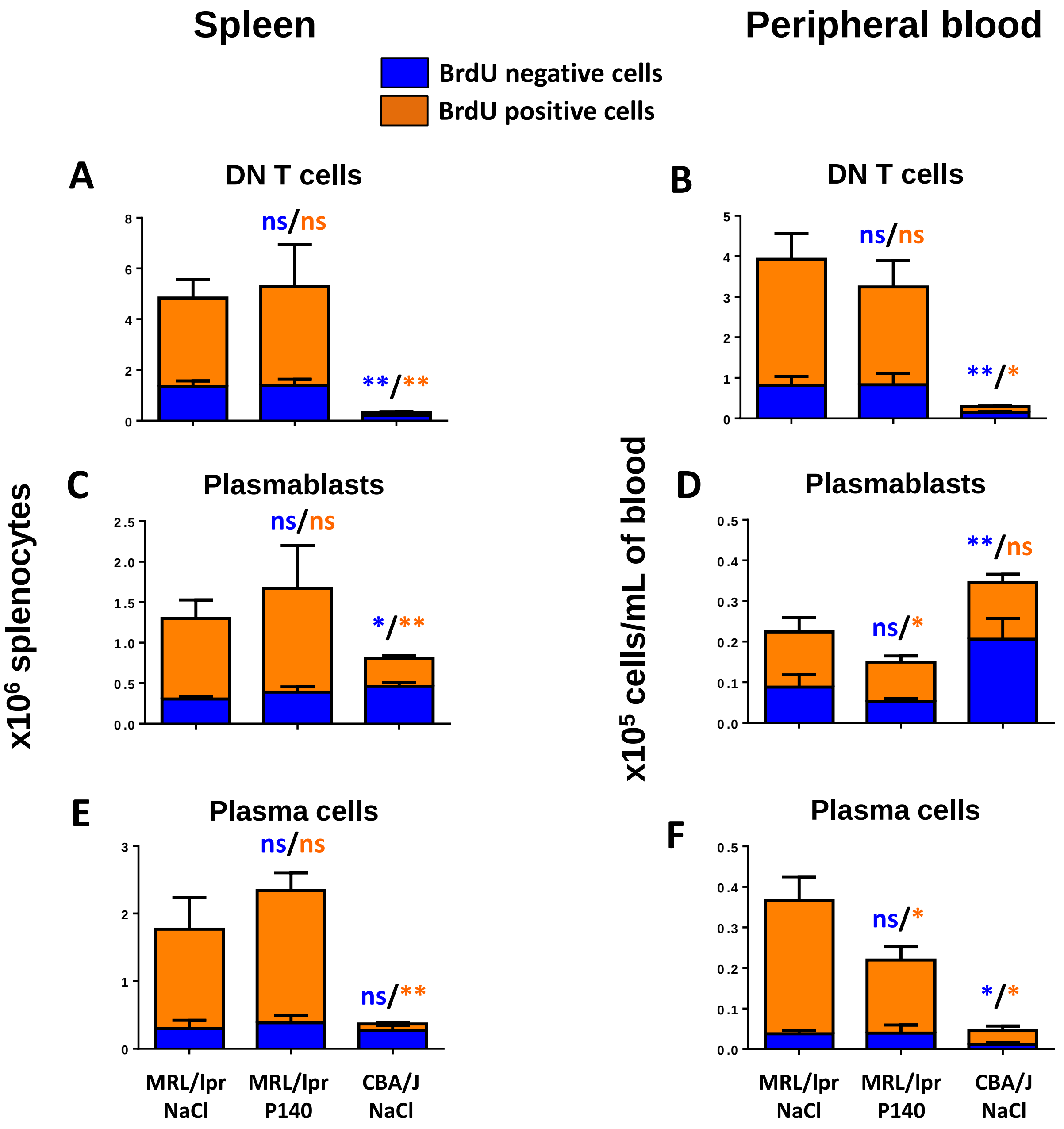

Supplementary Figure 2

Long-term *in vivo* BrdU incorporation in various immune cell subsets of CBA/J and MRL/lpr mice that received or not P140 peptide. Age-matched mice received each a first intraperitoneal injection of BrdU, which was then administrated in drinking water for 10 days. 10-11 week-old female MRL/lpr mice treated with P140 peptide were given a single i.v. dose of peptide 5 days after they received intraperitoneal injection of BrdU. Cells were collected from the spleen and PBMCs, stained by incubating them with fluorescently-tagged antibodies to appropriate surface markers, fixed stained with anti-BrdU antibodies, and analyzed by flow cytometry. Histograms represent the mean ± SD of BrdU $^+$ (cycling) or $^-$ (non-cycling) cells derived from 5 mice per group. Statistical significance was assessed using the Mann Whitney test. *p<0.05; **p<0.01; ns, non-significant.

Supplementary Figure 3

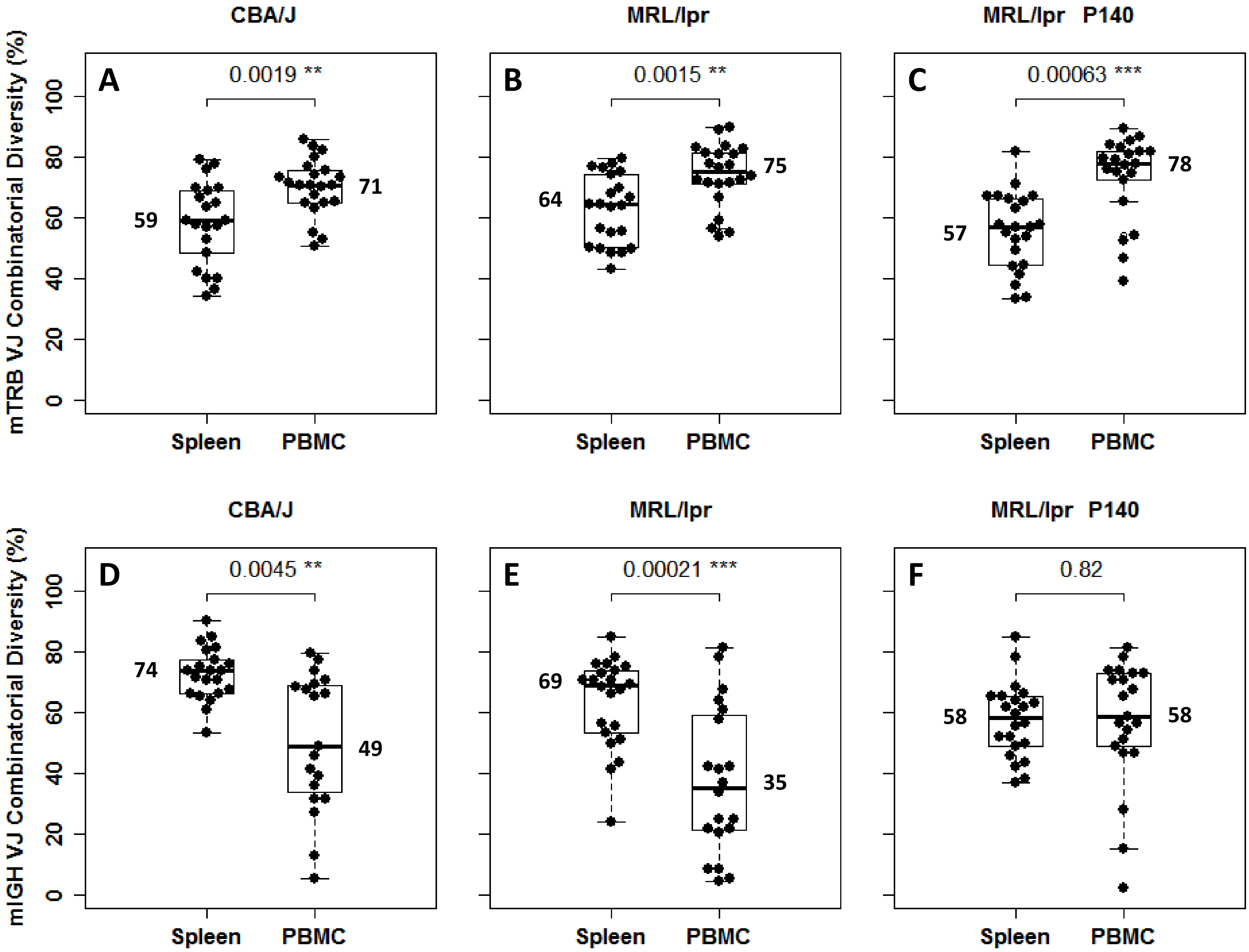

Supplementary Figure 3 Dot plot representation of mTRB VJ (A-C) and mIgH VJ (D-F) combinatorial diversity distribution in spleen and PBMC samples, for each group of mice. Horizontal lines represent median of diversity with corresponding values. Statistical significance was assessed using the Wilcoxon signed-rank test. P values are indicated.

The median mTRB diversity in spleen samples was 59% (range 34.5-78.9) and 64% (range 43.1-79.4) in CBA/J and MRL/lpr mice, respectively (p=0.380; Wilcoxon rank-sum test), and 71% (range 50.7-85.7) and 75% (range 53.6-90.0) in CBA/J and MRL/lpr mice, respectively (p=0.110; Wilcoxon rank-sum test) in PBMC samples. The median mIGH diversity in the spleen was 74% (range 53.3-90.2) and 69% (range 23.9-84.8) in CBA/J and MRL/lpr mice, respectively (p=0.055; Wilcoxon rank-sum test), and 49% (range 5.4-79.4) and 35% (range 4.4-81.5), respectively (p=0.0766; Wilcoxon rank-sum test) in PBMCs. It is worth noting that as expected due to the content of T and B cells in each compartment, the T cell population was significantly more diversified in the peripheral blood than in the spleen (p=0.0019 and 0.0015, respectively in CBA/J and MRL/lpr mice; Wilcoxon signed-rank test; Fig. S3, A and B) while, conversely, the B cell population was significantly more diversified in the spleen compared to the peripheral blood (p=0.0045 and 0.00021, respectively; Wilcoxon signed-rank test; Fig. S3, D and E). Since these observations were made both in CBA/J and MRL/lpr mice, the differences were considered as independent from lupus disease and/or genetic background.

As above, it was observed that the T cell population in P140-treated mice was more diversified in the peripheral blood than in the spleen (p= 0.00063; Wilcoxon signed-rank test; Fig. S3,C). Remarkably, mIGH VJ combinatorial diversity was statistically different in untreated and P140-treated MRL/lpr mice (p=0.047 and 0.012 in the spleen and PBMCs, respectively; Wilcoxon rank-sum test; Fig. 3, C and D). The median mIGH diversity in the spleen from treated mice was 58% (range 36.96-84.78) versus 69% (range 23.91-84.78) in MRL/lpr mice. In PBMCs, it was 58% (range 2.17-81.52) in treated mice versus 35% (range 4.35-81.52) in untreated mice. The B-cell population was equally diversified in the spleen and the peripheral blood (Fig. S3,F).

Supplementary Figure 4

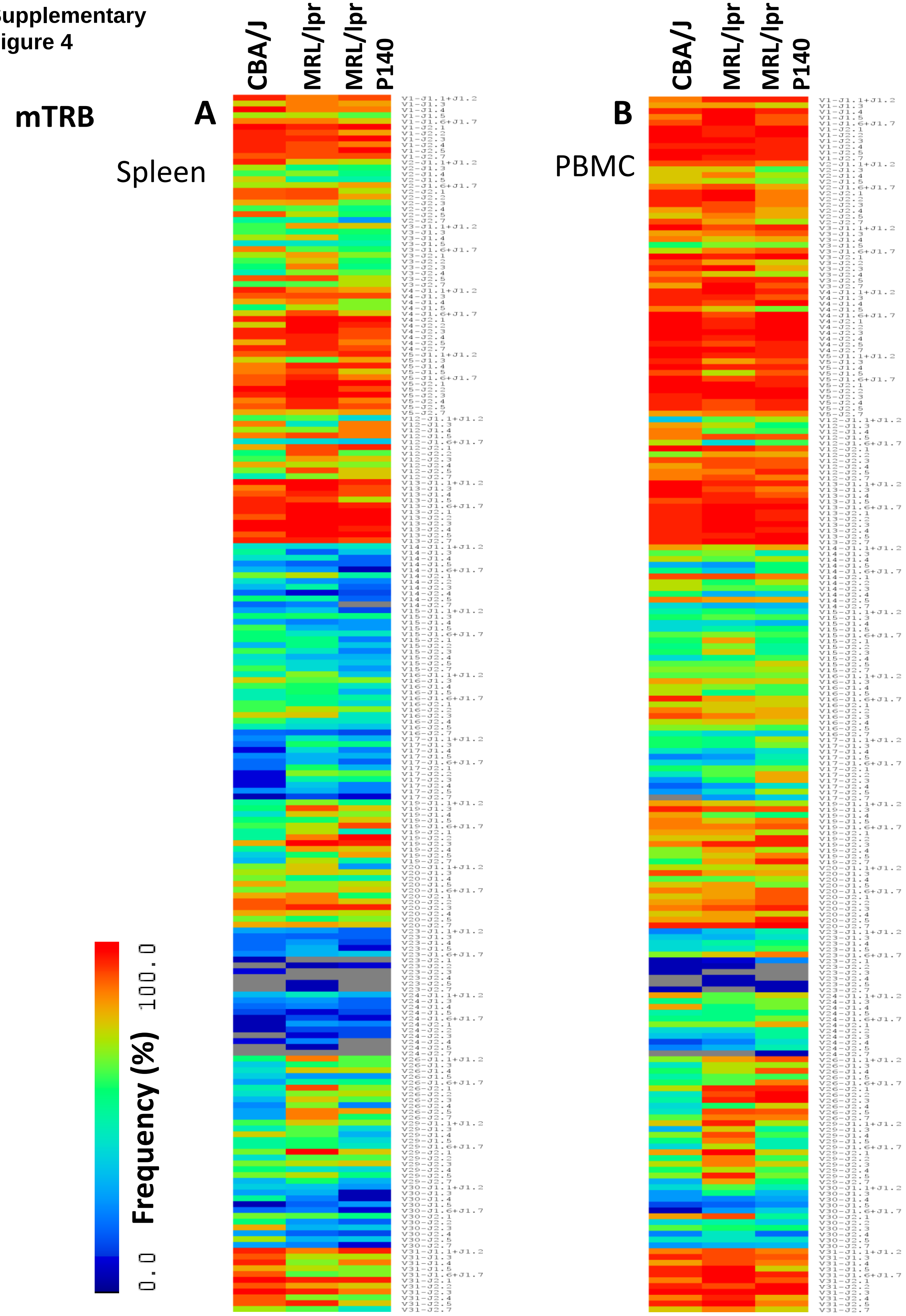

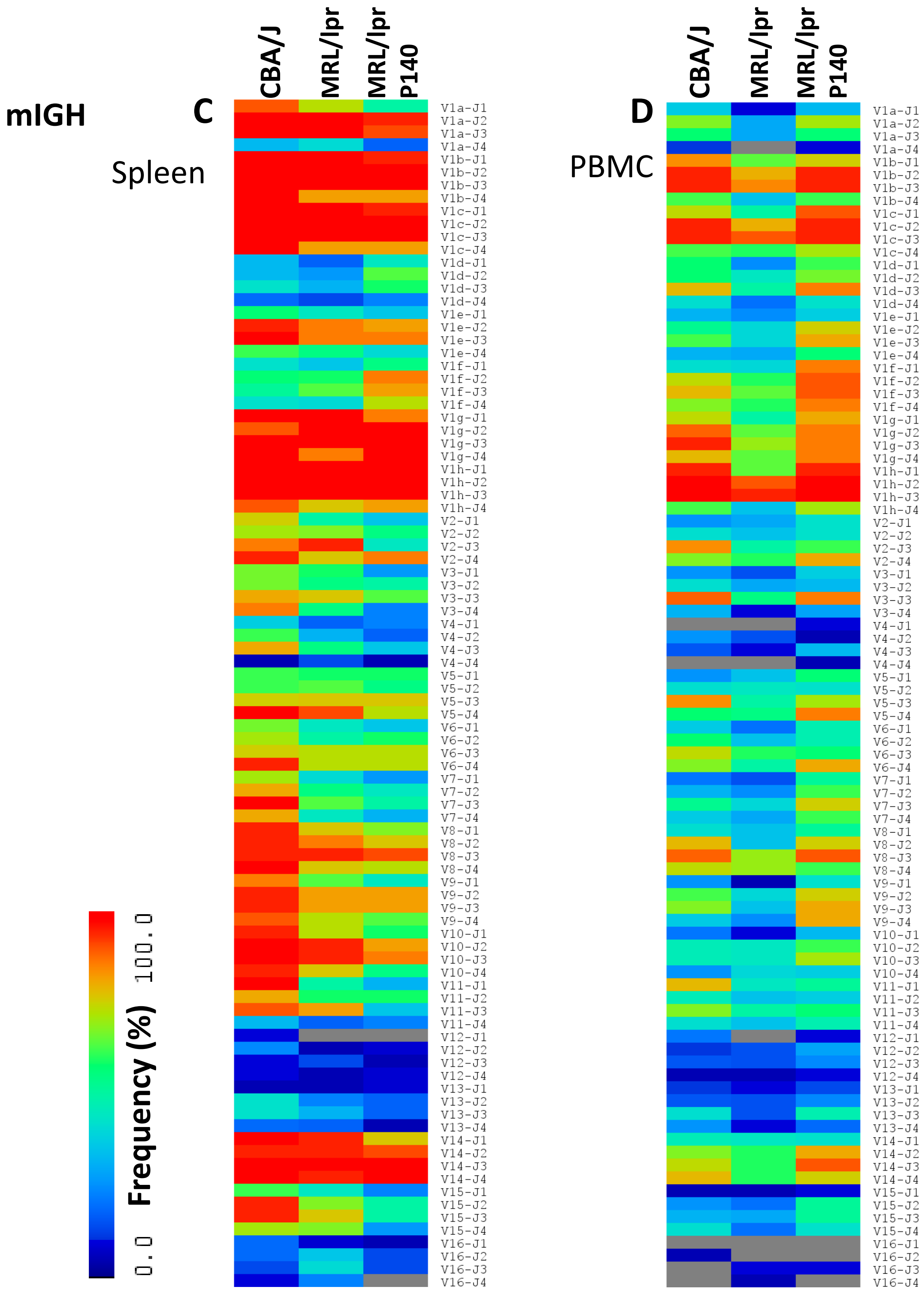
mIGH
C
Spleen
D
PBMC
CBA/J
MRL/lpr
MRL/lpr P140
Frequency (%)
0.0
100.0
V1a-J1
V1a-J2
V1a-J3
V1a-J4
V1b-J1
V1b-J2
V1b-J3
V1b-J4
V1c-J1
V1c-J2
V1c-J3
V1c-J4
V1d-J1
V1d-J2
V1d-J3
V1d-J4
V1e-J1
V1e-J2
V1e-J3
V1e-J4
V1f-J1
V1f-J2
V1f-J3
V1f-J4
V1g-J1
V1g-J2
V1g-J3
V1g-J4
V1h-J1
V1h-J2
V1h-J3
V1h-J4
V2-J1
V2-J2
V2-J3
V2-J4
V3-J1
V3-J2
V3-J3
V3-J4
V4-J1
V4-J2
V4-J3
V4-J4
V5-J1
V5-J2
V5-J3
V5-J4
V6-J1
V6-J2
V6-J3
V6-J4
V7-J1
V7-J2
V7-J3
V7-J4
V8-J1
V8-J2
V8-J3
V8-J4
V9-J1
V9-J2
V9-J3
V9-J4
V10-J1
V10-J2
V10-J3
V10-J4
V11-J1
V11-J2
V11-J3
V11-J4
V12-J1
V12-J2
V12-J3
V12-J4
V13-J1
V13-J2
V13-J3
V13-J4
V14-J1
V14-J2
V14-J3
V14-J4
V15-J1
V15-J2
V15-J3
V15-J4
V16-J1
V16-J2
V16-J3
V16-J4

Supplementary Figure 4 Overview of the frequency pattern for the 209 mTRB (A, B) and 92 mIGH (C, D) VJ theoretical rearrangements observed in spleen (A, C) and PBMC (B, D) samples from the indicated groups of mice. Colour code goes from dark blue to red, corresponding to the lowest and highest frequencies (in %); in grey the frequency is null.

Supplementary figure 5

A. mTRB Diversity

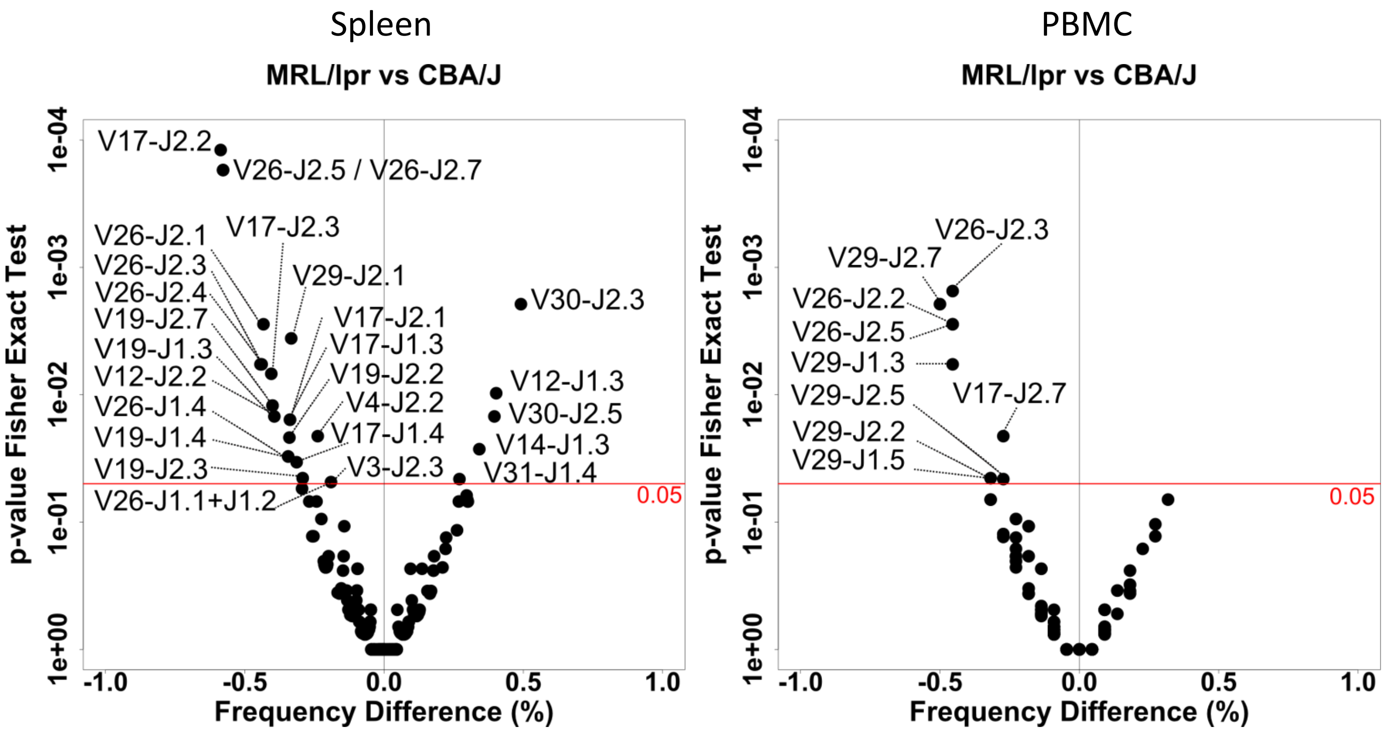

B. mIGH Diversity

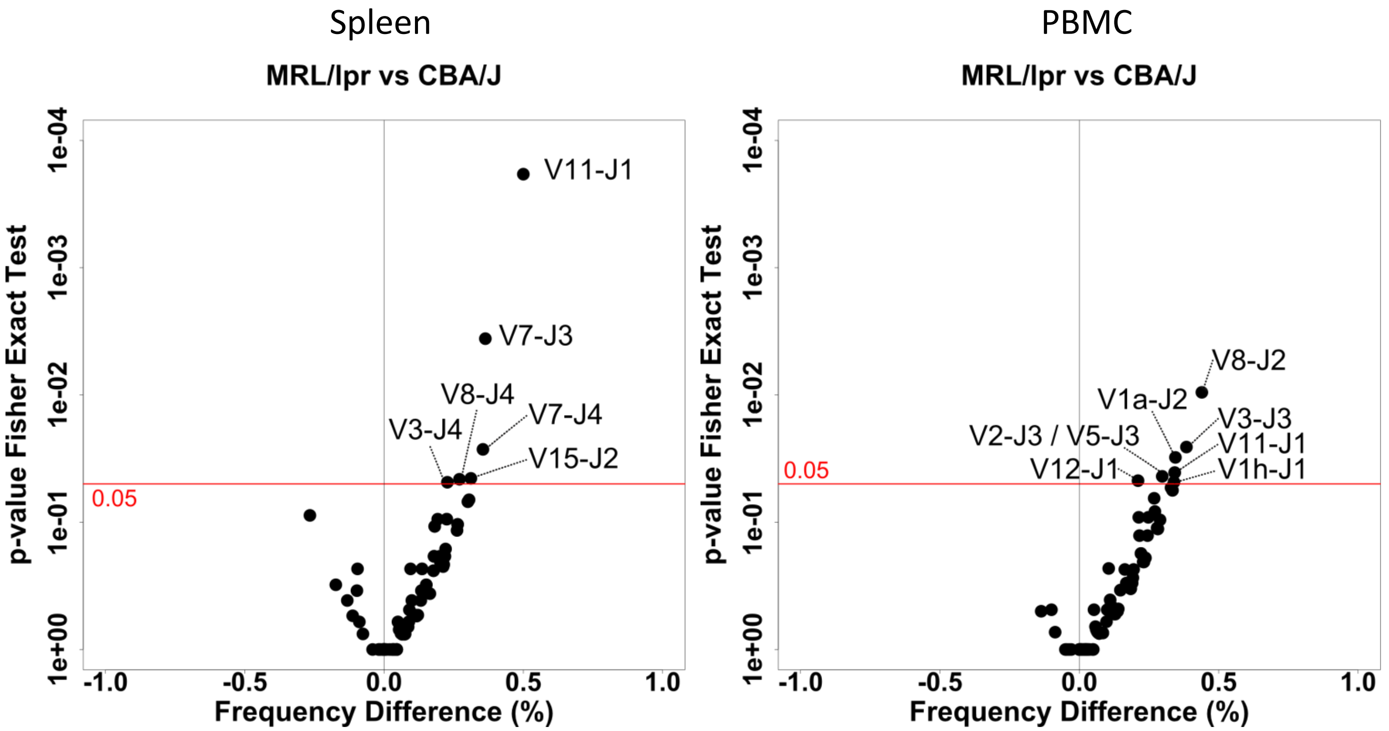

Supplementary Figure 5 Distribution of the significantly detected mTRB VJ (A) and mIGH (B) rearrangements in CBA/J and MRL/lpr mice. The analysis was made from spleen and PBMC samples. Statistically significant differences of rearrangements frequencies between the two groups of mice are shown. P values <0.05 (dots above the line) were considered significant by Fisher's exact test.

Supplementary Figure 6

A. mTRB Diversity

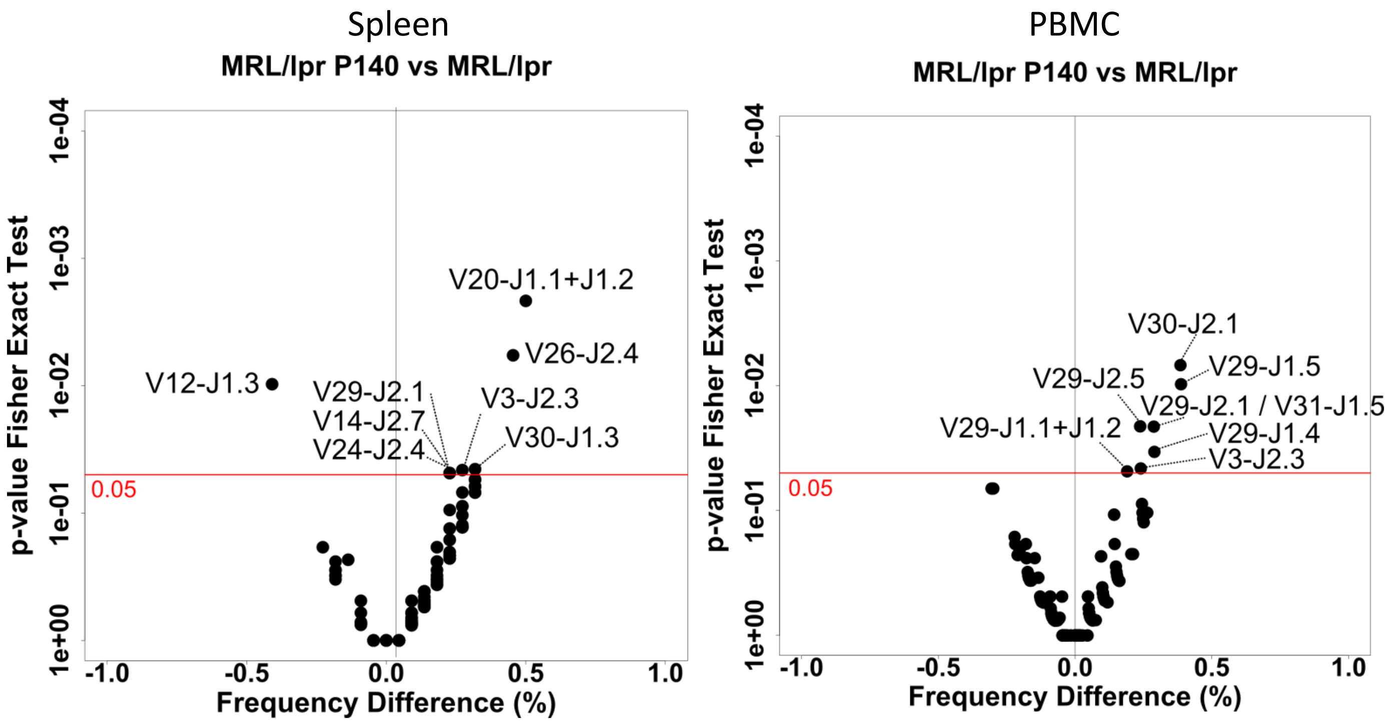

B. mIGH Diversity

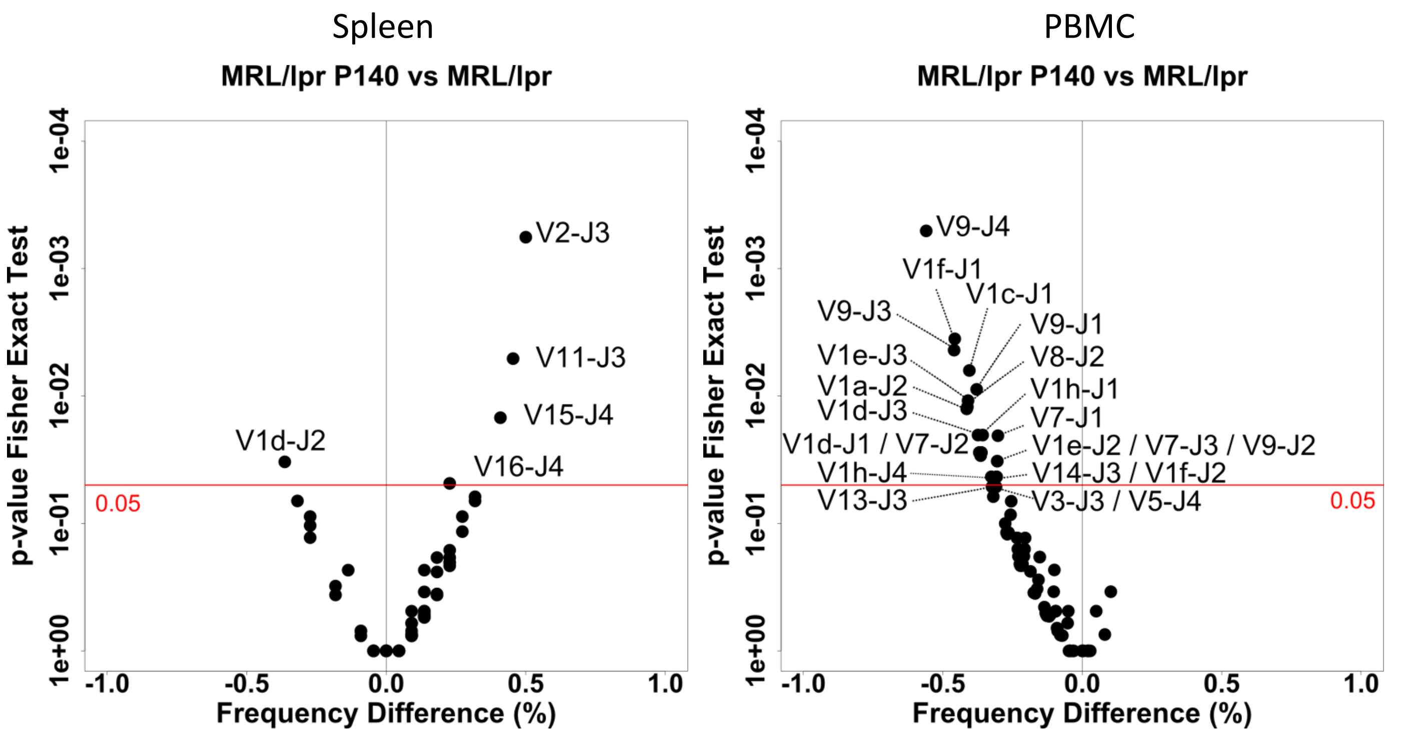

Supplementary Figure 6 Distribution of the significantly detected mTRB VJ (A) and mIGH (B) rearrangements in MRL/lpr and P140 treated-MRL/lpr mice. See the legend of Figure S5 for details.

Supplementary Figure 7

A. mTRB Diversity

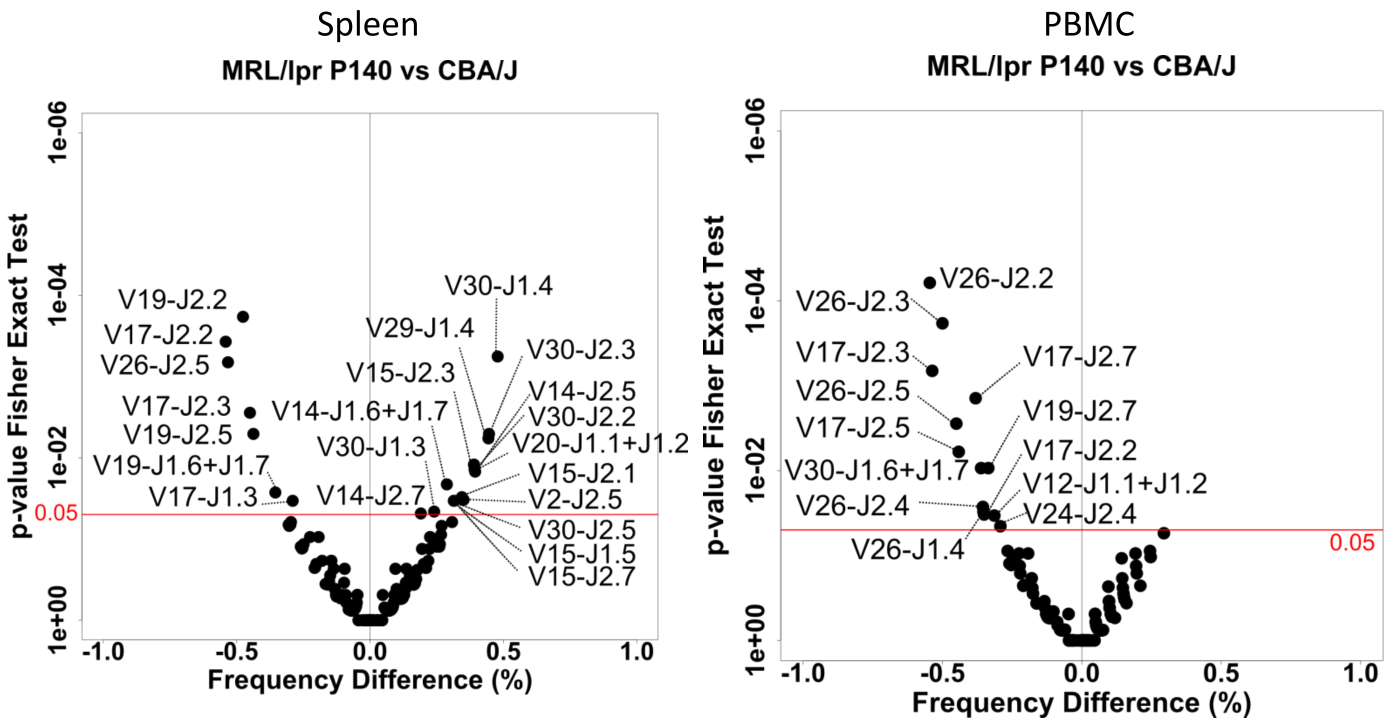

B. mIGH Diversity

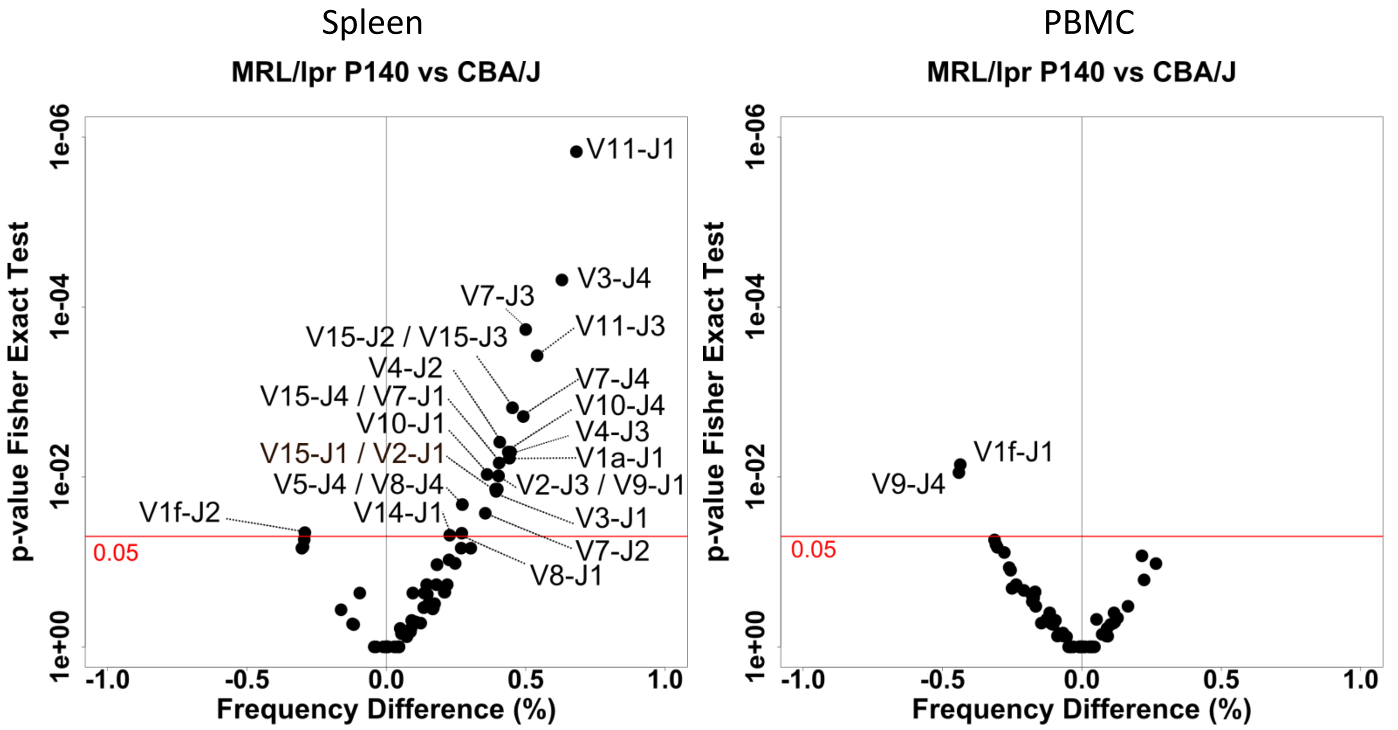

Supplementary Figure 7 Distribution of the significantly detected mTRB VJ (A) and mIGH (B) rearrangements in CBA/J and P140-treated MRL/lpr mice. See the legend of Figure S5 for details.

Panel B

Here are compared the mIGH VJ repertoire between CBA/J and P140-treated MRL/lpr mice (see also the Supplemental Table 5). All 6 IGH VJ rearrangements (V11-J1, V7-J3, V7-J4, V8-J4, V3-J4 and V15-J2) visualized in CBA/J splenocytes when compared to untreated MRL/lpr mice (see Supplemental Figure 5B) are still significantly more represented in CBA/J compared to P140-treated MRL/lpr mice, indicating that those rearrangements are related to CBA/J background. Since in our hands no mIGH VJ repertoire signature could be visualized, neither in splenic nor in peripheral blood MRL/lpr B cells (see Supplementary Figure 5), it was obviously not possible to investigate a potential effect of peptide on this specific repertoire.

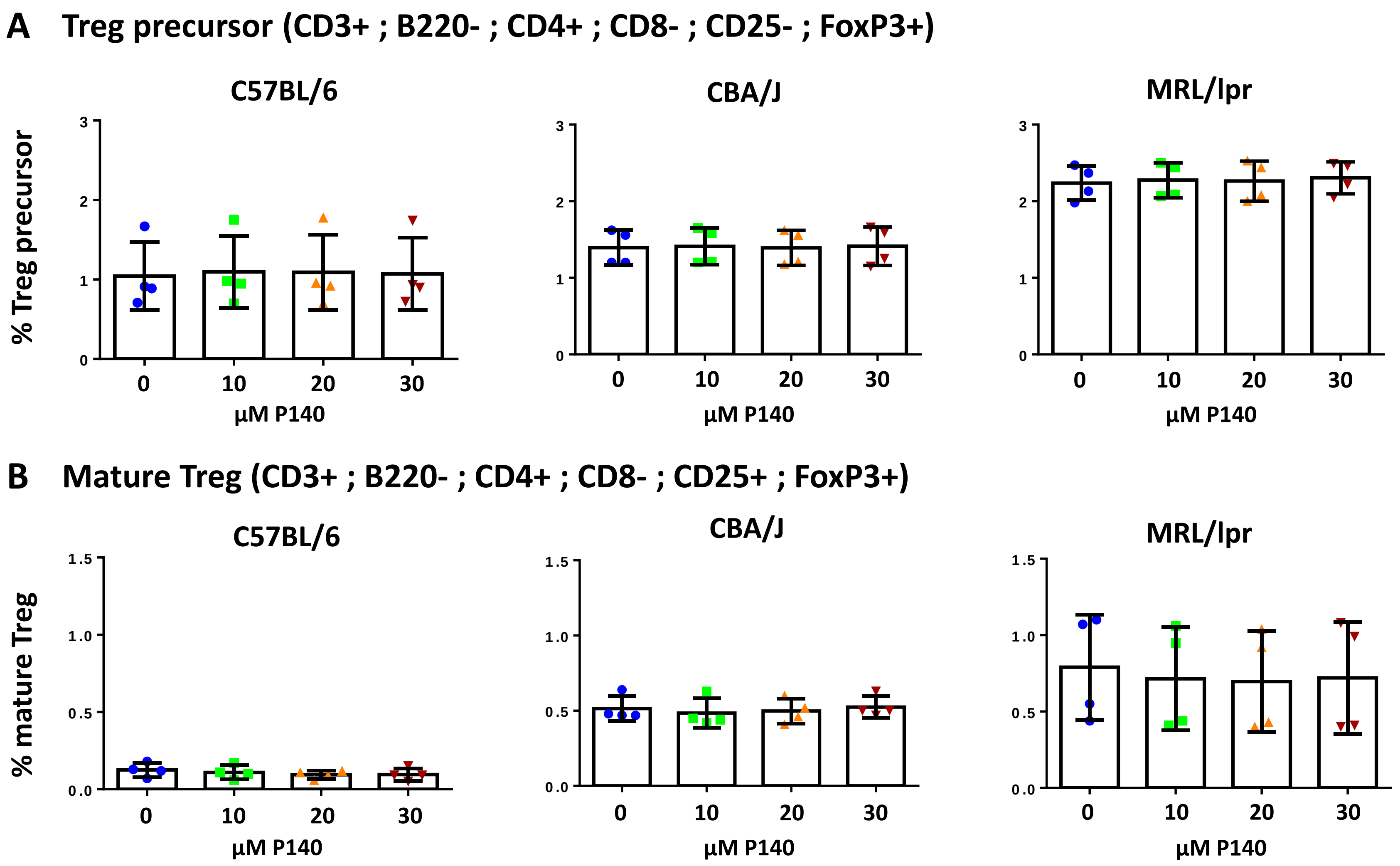

Supplementary Figure 8

In vitro effect of P140 on Tregs. Splenocytes from 11-13 week-old CBA/J, C57BL6 and MRL/lpr mice (two mice per strain) were separated using a 70 µm nylon mesh cell strainer. Red Blood cells were removed using AcK lysis buffer. Splenocytes were distributed in 24-well plates (2x10*6 cell per well) and incubated for 24h with increasing concentrations of P140 peptide as indicated. Splenocytes were then stained with the following antibodies (surface): CD3e PE-Cy7, CD45R/B220 eFluor506, CD4 AF700, CD8a APC-Cy7 and CD25 PerCP-Cy5 (refs. in the Method section). After washing, stained splenocytes were fixed, permeabilized using FoxP3 transcription factor staining buffer set (Invitrogen, ref. 00-5523-00) and intracellulary stained with FoxP3 FITC (Invitrogen, ref. 11-4776-42). Cells were analyzed using an Attune NxT cytometer from Invitrogen. Experiments were repeated independently twice.

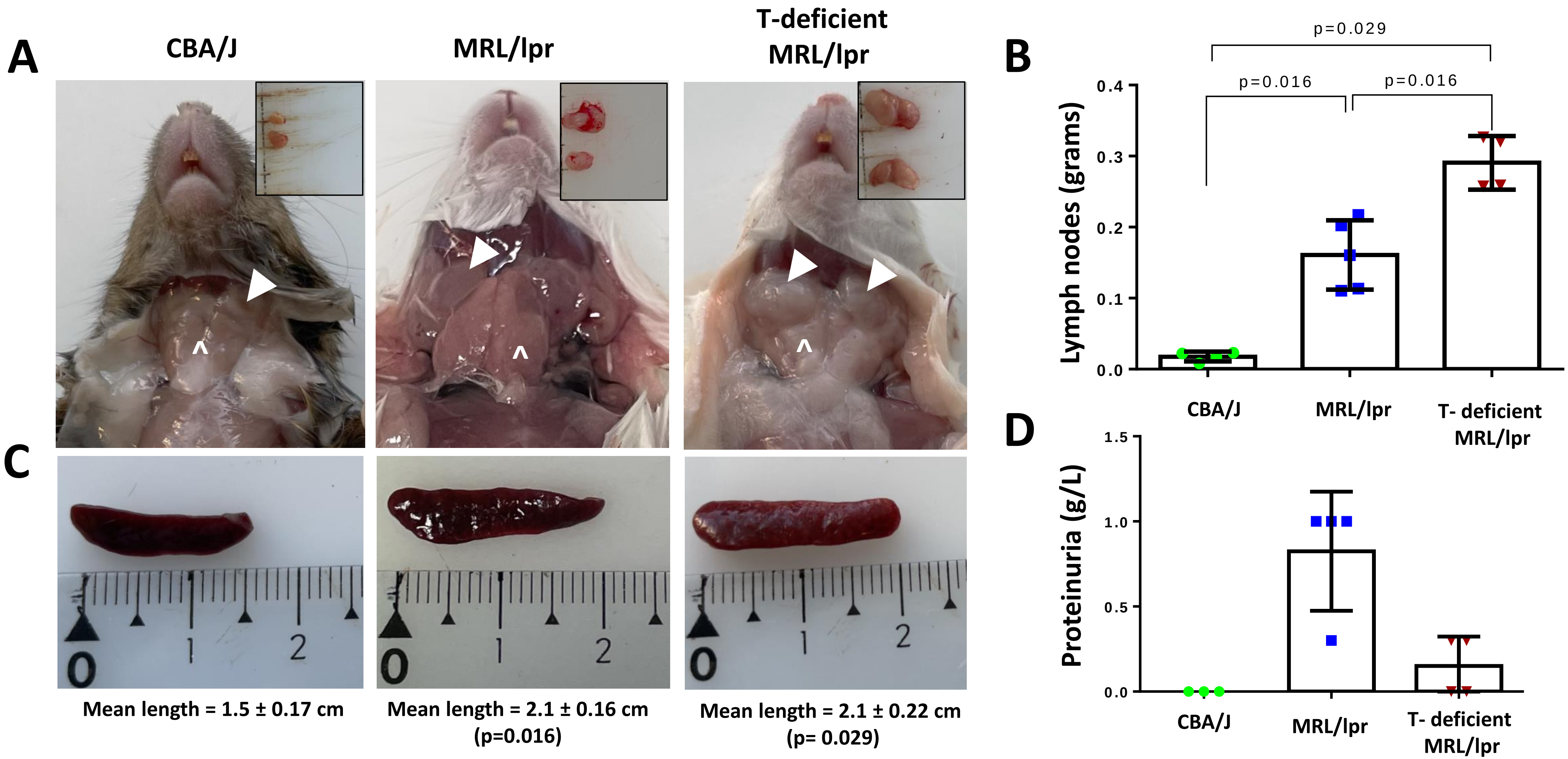

Supplementary Figure 9: Phenotypical comparison of healthy 1 year-old CBA/J mice with 13 week-old MRL/lpr strains. A. Representative pictures of parotid lymph nodes (△) and salivary glands (**Λ**). Insert: magnification of excided parotid lymph nodes. **B.** Weight (in grams) of lymph nodes (mean ± SD; n = 4/group). **C.** Spleen (representative picture and mean length ; n = 4 mice/group). **D.** Urine protein levels as assessed semi-quantitatively using albumin reagent strips. P values were calculated using Mann-Whitney test.

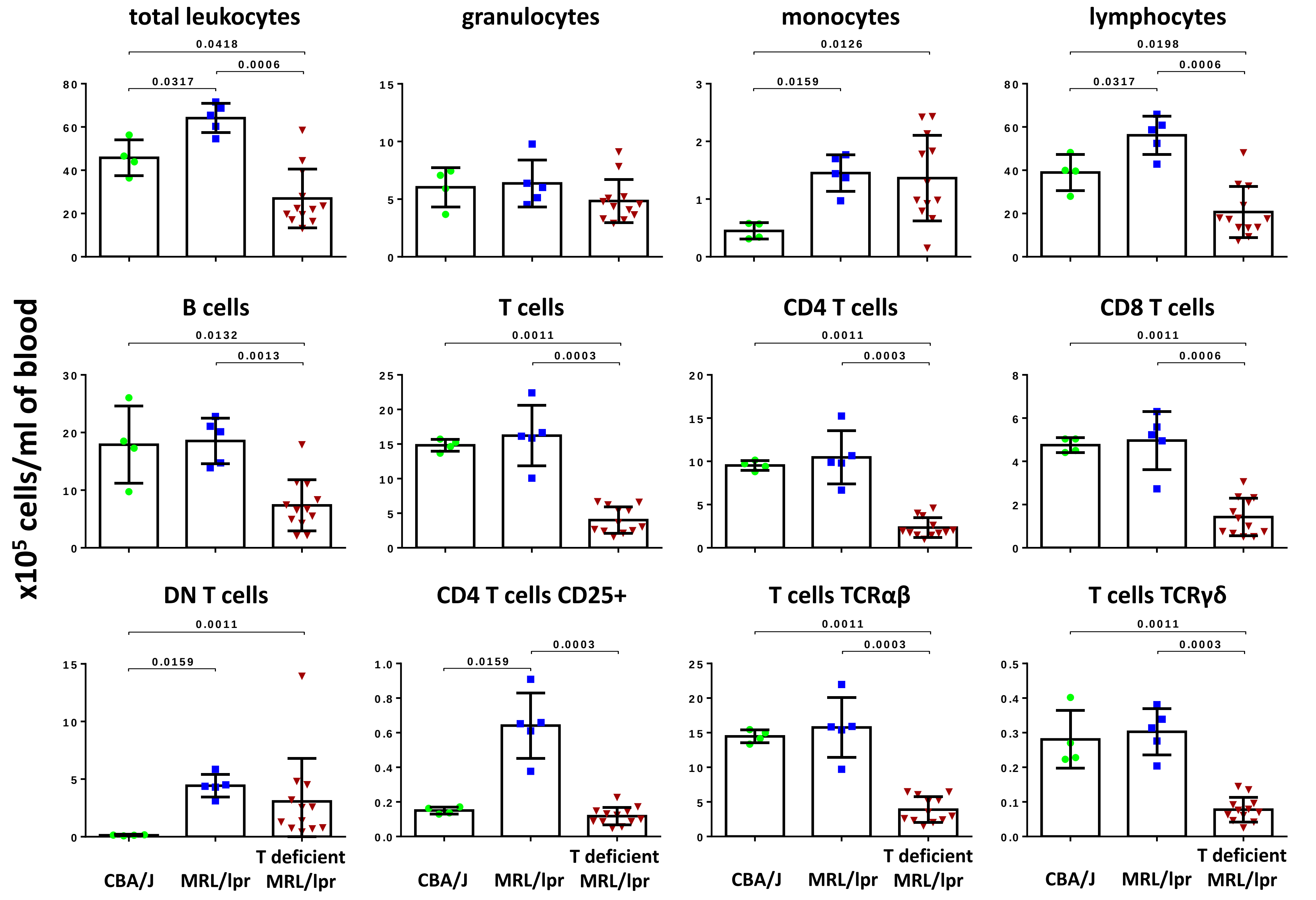

Supplementary Figure 10

Supplementary Figure 10

Phenotypes of immune cells present in the peripheral blood of healthy CBA/J mice and lupus-prone MRL/lpr mice. Cells collected from the blood of five 30 week-old CBA/J, five 13 week-old MRL/lpr mice and twelve 13 week old T-deficient MRL/lpr mice. After erythocyte lysis and washing, cells were washed with PBS 2% v/v fœtal bovine serum (FBS) and stained with LIVE/DEAD Fixable Violet SB436 for 30 mins at room temperature in the dark in PBS 2% FBS. To determine their phenotypes, leukocytes were washed with PBS 2% FBS and stained with the following antibodies (references in the Method section): TCRαβ-FITC, TCRγδ-PE, Ly6C-PerCP-Cy5.5, CD45-PE-Cy7, CD8a-APC, CD3-AF700, CD4-APC-Cy7, Gr-1-eFluor 506, CD25-SB600, B220-SB702. All antibodies were used at a 1µg/mL concentration except antibodies to CD45 used at 0.6µg/mL in 200µl PBS 2% FBS per well. After 30 mins of staining, cells were washed, resuspended in 200µL PBS 2% SVF and analyzed using an Attune NxT cytometer (Invitrogen). P values were calculated using Mann-Withney test. Significant P values only are indicated.
