# Supplementary material for: P140 Peptide Leads to Clearance of Autoreactive Lymphocytes and Normalizes Immune Response in Lupus-Prone Mice
Source: Front Immunol. 2022 Jun 1;13:904669. doi: 10.3389/fimmu.2022.904669 (PMC9199391; doi:10.3389/fimmu.2022.904669)
Supplement: Supplementary file 2 [file DataSheet_2.pdf]

frontiers

Supplementary Material

P140 peptide leads to clearance of autoreactive lymphocytes and normalizes immune response in lupus-prone mice

Nicolas Schall, Laura Talamini, Maud Wilhelm, Evelyne Jouvin-Marche,
and Sylviane Muller

1 Comparison of TCR and BCR V-J rearrangements in CBA/J and MRL/lpr mice

Before investigating the effect of P140, our first aim was to assess the extent of TCR and BCR repertoire impairment in MRL/lpr mice in comparison to CBA/J mice, which share the same MHC and murine T cell receptor β-chain (mTRB) haplotypes (1).

A detailed analysis of mTRB and mIGH diversity and combinatorial repertoire composition was performed from spleen and PBMC samples of CBA/J and MRL/lpr mice. We used a general immune companion diagnostic assay to monitor T and B cell responses and evaluate the immune statute in the different strains of mice (2). The results were compared for each group of mice and for each repertoire. A total of 43 spleen and 44 PBMC samples were analyzed for mTRB repertoire; 43 spleen and 39 PBMC samples were analyzed for mIGH repertoire (Table S1). When CBA/J and MRL/lpr mice samples were compared, no statistically significant difference was observed in terms of mTRB and mIGH combinatorial diversity (Figure 3; Supplementary Figures 3A, B).

For exploring both repertoires more deeply, analyses of VJ rearrangements were made based on the frequency of detection of each of the 209 mTRB and 92 mIGH theoretical rearrangements per group of mice (Supplementary Figures 4A-D). The frequency of detection was estimated through the number of times a given Vi-Jj rearrangement was detected and the number of samples in the concerned group. The analysis done on the entire mTRB VJ repertoire revealed that 5 mTRB VJ rearrangements in spleen were significantly represented in CBA/J mice when compared to MRL/lpr mice (Supplementary Figure 5A). mTRB VJ rearrangements (21 in spleen; 9 in PBMCs) were significantly more frequent in MRL/lpr mice compared to CBA/J mice (Supplementary Figures 5A; Supplementary Table 3). V26-J2.3 and V26-J2.5 rearrangements appearing significantly more frequently in MRL/lpr mice, and in

both the spleen and PBMCs, might represent a privileged combinatory VJ repertoire signature of this mouse strain. Thus, between CBA/J and MRL/lpr mice no statistically significant difference exists both in the spleen and PBMC fraction in terms of mTRB combinatorial diversity but some specific mTRB VJ rearrangements are statistically more frequently represented in MRL/lpr mice and could be designed as a signature.

A similar analysis done on the entire mIGH VJ repertoire revealed that besides common rearrangements, mIGH VJ rearrangements (6 in spleen; 8 in PBMCs) were significantly more frequent in CBA/J mice compared to MRL/lpr mice. None were found significantly more represented in MRL/lpr (Fig. S5; Table S3). Thus, no mIGH VJ repertoire signature could be highlighted in the MRL/lpr model with regard to CBA/J mice.

1.1 Effect of P140 on the frequency of TCR and BCR VJ rearrangements in MRL/lpr mice

The next step was to examine the possible influence of P140 on these rearrangements. Spleen and PBMC samples (Supplementary Table 1) collected 5 days post-administration of P140 were analyzed for mTRB and mIGH VJ repertoires. When untreated and P140-treated MRL/lpr mice were compared, no difference was observed in splenocytes and PBMCs in terms of mTRB VJ combinatorial diversity (Figures 3A-B; Supplementary Figures 3C-F).

The analysis done on the entire mTRB VJ repertoire (Supplementary Figure 4) revealed that compared to untreated MRL/lpr mice, one mTRB VJ rearrangement in spleen (none in PBMCs) was significantly more frequent in P140-treated mice (Supplementary Figure 6A; Supplementary Table 4). However, mTRB VJ rearrangements (7 in spleen, 8 in PBMCs) were found more frequently in untreated MRL/lpr mice compared to P140-treated mice. From those mTRB VJ rearrangements, V29-J2.1 and V3-J2.3, which were found both in splenocytes and PBMCs from untreated mice, could represent a signature that is absent in P140-treated mice. Regarding the fate of the mTRB VJ rearrangements distinguishing MRL/lpr spleen from CBA/J mice, some (V3-J2.3, V26-J2.4, V29-J2.1) also present a significant difference of frequency between treated and untreated MRL/lpr mice, meaning that they are apparently affected by the treatment. The analysis done with PBMC samples led to the same conclusions, which concern mTRB VJ rearrangements V29-J2.5 and V29-J1.5. These findings collectively suggest that pre- and post-P140 treatment, the distribution of frequencies of some mTRB VJ rearrangements is changed. To reinforce this assumption, the repertoire composition of the treated MRL/lpr mice was compared to the CBA/J control one.

The repertoire comparison showed that mTRB VJ rearrangements (15 in spleen but none in PBMCs) were significantly more represented in CBA/J while 7 in spleen and 13 in PBMCs could be considered as specifically related to the treated MRL/lpr repertoire (Supplementary Figure 7A; Supplementary Table 5). In the list of mTRB VJ rearrangements, which might putatively sign MRL/lpr features (Supplementary Figure 5A), V26-J2.5, V17-J2.2 and V17-J2.3 rearrangements were found highly represented both in the spleen and PBMCs of P140-treated MRL/lpr mice compared to CBA/J mice. However, no significant difference of frequency for these rearrangements was found between P140-treated and untreated MRL/lpr mice (Supplementary Figure 6A), indicating that P140 may have no influence on these rearrangements. However, regarding the other identified rearrangements (V3-J2.3, V26-J2.4, and V29-J2.1 in spleen and V29-J2.5 and V29-J1.5 in PBMCs), frequencies dropped in P140-treated mice to values close to the ones found in the CBA/J mice (no significant difference of frequency could be highlighted for these rearrangements between the two groups; Supplementary Figure 7A). Taken together, these results indicate that treating MRL/lpr mice with P140 impacts the distribution of the frequency of rare, MRL/lpr-related mTRB VJ rearrangements, which reach levels that are similar to those found in healthy CBA/J mice.

The comparison of mIGH VJ repertoires of MRL/lpr mice treated or not with P140 (Supplementary Figures 4C-D) revealed that more frequent differences occur in PBMCs than in spleens, where 22 rearrangements were found significantly more often in P140-treated than in untreated MRL/lpr mice (Supplementary Figures 6B; Supplementary Table 4). Thus, as found in the case of mTRB VJ rearrangements, the distribution of the frequency of mIGH VJ rearrangements also seems to be affected by P140 treatment. As above in the case of mTRB VJ repertoire, we compared the mIGH VJ repertoire between CBA/J and P140-treated MRL/lpr mice (see details in Supplementary Figure 7B; Supplementary Table 5). Since in our hands no mIGH VJ repertoire signature could be visualized in MRL/lpr B cells, it was not possible to investigate a potential effect of peptide on this specific repertoire.

References

1. Baum T-P, Pasqual N, Thuderoz F, Hierle V, Chaume D, Lefranc M-P, Jouvin-Marche E, Marche P-N, Demongeot J. IMGT/GeneInfo: enhancing V(D)J recombination database accessibility. *Nucleic Acids Res* (2004) **32**:D51-54. doi: 10.1093/nar/gkh041

2. Pasqual N, Gallagher M, Aude-Garcia C, Loiodice M, Thuderoz F, Demongeot J, Ceredig R, Marche PN, Jouvin-Marche E. Quantitative and qualitative changes in V-J alpha rearrangements

during mouse thymocytes differentiation: implication for a limited T cell receptor alpha chain repertoire. *J Exp Med* (2002) **196**:1163–1173. doi: 10.1084/jem.20021074
