# Supplementary material for: P140 Peptide Leads to Clearance of Autoreactive Lymphocytes and Normalizes Immune Response in Lupus-Prone Mice
Source: Front Immunol. 2022 Jun 1;13:904669. doi: 10.3389/fimmu.2022.904669 (PMC9199391; doi:10.3389/fimmu.2022.904669)
Supplement: Supplementary file 3 [file Table_1.pdf]

mTRB repertoire

Mice	Treatment		Spleen samples	PBMC samples
	NaCl	P140		
CBA/J	X		21	22
MRL/lpr	X		22	22
MRL/lpr	X	X	22	21
Total:			**65**	**65**

mIGH repertoire

CBA/J	X		21	19
MRL/lpr	X		22	20
MRL/lpr	X	X	22	21
Total:			**65**	**60**

Supplementary Table 1: Description of the composition of each group of mice, their respective treatment and number of analyzed samples.

Abbreviations: CBA/J, C.C.x Bagg, strain A/Jackson; lpr, lymphoproliferation; mIGH, murine Ig heavy-chain; MRL, Murphy Roths large; PBMCs, peripheral blood mononuclear cells; mTRB, murine T cell receptor β-chain.

Groups	mTRB VJ rearrangements significantly detected (theoretical = 209)				mIGH V rearrangements significantly detected (theoretical = 92)			
	Spleen		PBMC		Spleen		PBMC	
	N	n	N	n	N	n	N	n
**CBA/J NaCl**	21	**75**	22	**112**	21	**55**	19	**20**
**MRL/lpr NaCl**	22	**82**	22	**127**	22	**40**	20	**6**
**MRL/lpr NaCl + P140**	22	**70**	21	**119**	22	**34**	21	**33**

Supplementary Table 2: Number (N) of significantly detected mTRB and mIGH VJ rearrangements for each tested group found over the theoretical rearrangements.
For each tested group of mice the number of samples (n) is indicated.

Abbreviations: CBA/J, C.C.x Bagg, strain A/Jackson; lpr, lymphoproliferation; mIGH, murine Ig heavy-chain; MRL, Murphy Roths large; PBMCs, peripheral blood mononuclear cells; mTRB, murine T cell receptor β-chain; V, variable.

Supplementary Table 3

A. mTRB Diversity

mTRB VJ rearrangement/Spleen	Frequency Detection in the group CBA/J (N=21)	MRL/lpr (N=22)	Frequency Difference	Fisher Exact Test p-value
**V30-J2.3**	81%	32%	49%	0,0019
**V12-J1.3**	86%	45%	40%	0,0097
**V30-J2.5**	71%	32%	40%	0,0148
**V14-J1.3**	52%	18%	34%	0,0268
**V31-J1.4**	95%	68%	27%	0,0459
**V19-J2.3**	81%	100%	-19%	0,0485
**V4-J2.2**	76%	100%	-24%	0,0211
**V26-J1.1+J1.2**	57%	86%	-29%	0,0452
**V3-J2.3**	57%	86%	-29%	0,0452
**V17-J1.4**	10%	41%	-31%	0,0339
**V29-J2.1**	67%	100%	-33%	0,0036
**V12-J2.2**	57%	91%	-34%	0,0157
**V19-J1.3**	57%	91%	-34%	0,0157
**V19-J2.2**	52%	86%	-34%	0,0217
**V19-J1.4**	43%	77%	-34%	0,0305
**V26-J1.4**	43%	77%	-34%	0,0305
**V19-J2.7**	33%	73%	-39%	0,0148
**V17-J1.3**	19%	59%	-40%	0,0122
**V17-J2.1**	19%	59%	-40%	0,0122
**V17-J2.3**	10%	50%	-40%	0,0068
**V26-J2.1**	48%	91%	-43%	0,0028
**V26-J2.3**	33%	77%	-44%	0,0058
**V26-J2.4**	24%	68%	-44%	0,0058
**V26-J2.5**	29%	86%	-58%	2,00E-04
**V26-J2.7**	29%	86%	-58%	2,00E-04
**V17-J2.2**	10%	68%	-59%	1,00E-04

mTRB VJ rearrangement/PBMC	Frequency Detection in the group CBA/J (N=22)	MRL/lpr (N=22)	Frequency Difference	Fisher Exact Test p-value
**V17-J2.7**	0%	27%	-27%	0,021
**V29-J2.5**	68%	96%	-27%	0,046
**V29-J1.5**	55%	86%	-32%	0,045
**V29-J2.2**	55%	86%	-32%	0,045
**V26-J2.3**	50%	96%	-45%	0,002
**V26-J2.2**	45%	91%	-45%	0,003
**V26-J2.5**	45%	91%	-45%	0,003
**V29-J1.3**	32%	77%	-45%	0,006
**V29-J2.7**	32%	82%	-50%	0,002

B. mIGH Diversity

mIGH VJ rearrangement/Spleen	Frequency Detection in the group			
	CBA/J (N=21)	MRL/lpr (N=22)	Frequency Difference	Fisher Exact Test p-value
**V11-J1**	100%	50%	50%	2,00E-04
**V7-J3**	100%	64%	36%	0,0036
**V7-J4**	81%	45%	36%	0,0268
**V3-J4**	86%	55%	31%	0,0452
**V15-J2**	95%	68%	27%	0,0459
**V8-J4**	100%	77%	23%	0,0485

mIGH VJ rearrangement/PBMC	Frequency Detection in the group			
	CBA/J (N=19)	MRL/lpr (N=20)	Frequency Difference	Fisher Exact Test p-value
**V8-J2**	79%	35%	44%	0,0095
**V1a-J2**	68%	30%	38%	0,0256
**V3-J3**	89%	55%	34%	0,031
**V2-J3**	84%	50%	34%	0,0407
**V5-J3**	84%	50%	34%	0,0407
**V11-J1**	79%	45%	34%	0,0484
**V1h-J1**	95%	65%	30%	0,0436
**V12-J1**	21%	0%	21%	0,0471

Supplementary Table 4

A. mTRB Diversity

mTRB VJ rearrangement/spleen	Frequency Detection in the group MRL/lpr (N=22)	MRL/lpr P140 (N=22)	Frequency Difference	Fisher Exact Test p-value
**V20-J1.1+J1.2**	77%	27%	50%	0,0022
**V26-J2.4**	68%	23%	45%	0,0058
**V3-J2.3**	86%	55%	32%	0,0452
**V30-J1.3**	32%	5%	27%	0,0459
**V29-J2.1**	100%	77%	23%	0,0485
**V14-J2.7**	23%	0%	23%	0,0485
**V24-J2.4**	23%	0%	23%	0,0485
**V12-J1.3**	45%	86%	-41%	0,0097

mTRB VJ rearrangement/PBMC	Frequency Detection in the group MRL/lpr (N=22)	MRL/lpr P140 (N=21)	Frequency Difference	Fisher Exact Test p-value
**V29-J1.5**	86%	48%	39%	0,01
**V30-J2.1**	91%	52%	39%	0,007
**V29-J1.4**	91%	62%	29%	0,034
**V29-J2.5**	96%	67%	29%	0,021
**V29-J1.1+J1.2**	96%	71%	24%	0,046
**V29-J2.1**	100%	76%	24%	0,021
**V31-J1.5**	100%	76%	24%	0,021
**V3-J2.3**	100%	81%	19%	0,048

B. mIGH Diversity

mIGH VJ rearrangement/Spleen	Frequency Detection in the group			
	MRL/lpr (N=22)	MRL/lpr P140 (N=22)	Frequency Difference	Fisher Exact Test p-value
**V2-J3**	95%	45%	50%	6,00E-04
**V11-J3**	82%	36%	45%	0,0051
**V15-J4**	68%	27%	41%	0,0148
**V16-J4**	23%	0%	23%	0,0485
**V1d-J2**	27%	64%	-36%	0,0329

mIGH VJ rearrangement/PBMC	Frequency Detection in the group			
	MRL/lpr (N=20)	MRL/lpr P140 (N=21)	Frequency Difference	Fisher Exact Test p-value
**V1h-J1**	65%	95%	-30%	0,0205
**V14-J3**	60%	90%	-30%	0,0325
**V1f-J2**	60%	90%	-30%	0,0325
**V3-J3**	55%	86%	-31%	0,0431
**V5-J4**	55%	86%	-31%	0,0431
**V13-J3**	15%	48%	-33%	0,0431
**V1d-J3**	50%	86%	-36%	0,0203
**V1e-J2**	40%	76%	-36%	0,0278
**V7-J3**	40%	76%	-36%	0,0278
**V9-J2**	40%	76%	-36%	0,0278
**V1h-J4**	35%	71%	-36%	0,0294
**V1d-J1**	25%	62%	-37%	0,0278
**V7-J2**	25%	62%	-37%	0,0278
**V7-J1**	15%	52%	-37%	0,0203
**V9-J1**	5%	43%	-38%	0,0089
**V1c-J1**	50%	90%	-40%	0,0063
**V1e-J3**	40%	81%	-41%	0,0109
**V8-J2**	35%	76%	-41%	0,0122
**V1a-J2**	30%	71%	-41%	0,0126
**V1f-J1**	40%	86%	-46%	0,0036
**V9-J3**	35%	81%	-46%	0,0044
**V9-J4**	25%	81%	-56%	5,00E-04

Supplementary Table 5

A. mTRB Diversity

mTRB VJ rearrangement/Spleen	Frequency Detection in the group			
	CBA/J (N=21)	MRL/lpr P140 (N=22)	Frequency Difference	Fisher Exact Test p-value
**V30-J1.4**	52%	5%	48%	6,00E-04
**V30-J2.3**	81%	36%	45%	0,0051
**V29-J1.4**	76%	32%	44%	0,0058
**V20-J1.1+J1.2**	67%	27%	39%	0,0148
**V15-J2.3**	62%	23%	39%	0,0139
**V14-J2.5**	57%	18%	39%	0,0122
**V30-J2.2**	57%	18%	39%	0,0122
**V30-J2.5**	71%	36%	35%	0,0329
**V15-J1.5**	62%	27%	35%	0,0329
**V15-J2.7**	62%	27%	35%	0,0329
**V15-J2.1**	57%	23%	34%	0,0305
**V2-J2.5**	90%	59%	31%	0,0339
**V14-J1.6+J1.7**	33%	5%	29%	0,0212
**V30-J1.3**	29%	5%	24%	0,0459
**V14-J2.7**	19%	0%	19%	0,0485
**V19-J1.6+J1.7**	62%	91%	-29%	0,0339
**V17-J1.3**	19%	55%	-36%	0,0268
**V19-J2.5**	38%	82%	-44%	0,0051
**V17-J2.3**	10%	55%	-45%	0,0028
**V19-J2.2**	52%	100%	-48%	2,00E-04
**V26-J2.5**	29%	82%	-53%	7,00E-04
**V17-J2.2**	10%	64%	-54%	4,00E-04

mTRB VJ rearrangement/PBMC	Frequency Detection in the group			
	CBA/J (N=22)	MRL/lpr P140 (N=21)	Frequency Difference	Fisher Exact Test p-value
**V24-J2.4**	14%	43%	-29%	0,045
**V26-J1.4**	59%	91%	-31%	0,034
**V30-J1.6+J1.7**	5%	38%	-34%	0,009
**V12-J1.1+J1.2**	36%	71%	-35%	0,033
**V26-J2.4**	41%	76%	-35%	0,031
**V17-J2.2**	46%	81%	-36%	0,027
**V19-J2.7**	59%	95%	-36%	0,009
**V17-J2.7**	0%	38%	-38%	0,001
**V17-J2.5**	27%	71%	-44%	0,006
**V26-J2.5**	46%	91%	-45%	0,003
**V26-J2.3**	50%	100%	-50%	0,000
**V17-J2.3**	27%	81%	-54%	0,001
**V26-J2.2**	46%	100%	-55%	0,000

B. mIGH Diversity

mIGH VJ rearrangement/Spleen	Frequency Detection in the group CBA/J (N=21)	MRL/lpr P140 (N=22)	Frequency Difference	Fisher Exact Test p-value
**V11-J1**	100%	32%	68%	0,0000
**V3-J4**	86%	23%	63%	0,0000
**V11-J3**	90%	36%	54%	4,00E-04
**V7-J3**	100%	50%	50%	2,00E-04
**V7-J4**	81%	32%	49%	0,0019
**V15-J2**	95%	50%	45%	0,0015
**V15-J3**	95%	50%	45%	0,0015
**V4-J3**	81%	36%	45%	0,0051
**V15-J4**	71%	27%	44%	0,006
**V7-J1**	71%	27%	44%	0,006
**V4-J2**	62%	18%	44%	0,0051
**V10-J4**	95%	55%	41%	0,0039
**V1a-J1**	90%	50%	40%	0,0068
**V2-J3**	86%	45%	40%	0,0097
**V9-J1**	86%	45%	40%	0,0097
**V2-J1**	76%	36%	40%	0,0139
**V3-J1**	67%	27%	39%	0,0148
**V15-J1**	62%	23%	39%	0,0139
**V10-J1**	95%	59%	36%	0,0093
**V7-J2**	81%	45%	36%	0,0268
**V8-J4**	100%	73%	27%	0,0211
**V5-J4**	100%	73%	27%	0,0211
**V8-J1**	95%	68%	27%	0,0459
**V14-J1**	100%	77%	23%	0,0485
**V1f-J2**	57%	86%	-29%	0,0452

mIGH VJ rearrangement/PBMC	Frequency Detection in the group CBA/J (N=19)	MRL/lpr P140 (N=21)	Frequency Difference	Fisher Exact Test p-value
**V1f-J1**	42%	86%	-44%	0,0072
**V9-J4**	37%	81%	-44%	0,0089
